# The FHA domain protein ArnA functions as a global DNA damage response repressor in the hyperthermophilic archaeon *Saccharolobus islandicus*

**DOI:** 10.1128/mbio.00942-23

**Published:** 2023-06-30

**Authors:** Zhichao Jiang, Zijia Lin, Qi Gan, Pengju Wu, Xuemei Zhang, Yuanxi Xiao, Qunxin She, Jinfeng Ni, Yulong Shen, Qihong Huang

**Affiliations:** 1 CRISPR and Archaea Biology Research Center, State Key Laboratory of Microbial Technology, Microbial Technology Institute, Shandong University, Qingdao, China; Harvard Medical School, Boston, Massachusetts, USA

**Keywords:** FHA domain, archaea, DNA damage response, protein phosphorylation, signal transduction, transcription regulation

## Abstract

Forkhead-associated (FHA) domain proteins specifically recognize phosphorylated threonine via the FHA domain and are involved in signal transduction in various processes especially DNA damage response (DDR) and cell cycle regulation in eukaryotes. Although FHA domain proteins are found in prokaryotes, archaea, and bacteria, their functions are far less clear as compared to the eukaryotic counterparts, and it has not been studied whether archaeal FHA proteins play a role in DDR. Here, we have characterized an FHA protein from the hyperthermophilic Crenarchaeon *Saccharolobus islandicus* (SisArnA) by genetic, biochemical, and transcriptomic approaches. We find that Δ*SisarnA* exhibits higher resistance to DNA damage agent 4-nitroquinoline 1-oxide (NQO). The transcription of *ups* genes, encoding the proteins for pili-mediated cell aggregation and cell survival after DDR, is elevated in Δ*SisarnA*. The interactions of SisArnA with two predicted partners, SisvWA1 (SisArnB) and SisvWA2 (designated as SisArnE), were enhanced by phosphorylation *in vitro*. Δ*SisarnB* displays higher resistance to NQO than the wild type. In addition, the interaction between SisArnA and SisArnB, which is reduced in the NQO-treated cells, is indispensable for DNA binding *in vitro*. These indicate that SisArnA and SisArnB work together to inhibit the expression of *ups* genes *in vivo*. Interestingly, Δ*SisarnE* is more sensitive to NQO than the wild type, and the interaction between SisArnA and SisArnE is strengthened after NQO treatment, suggesting a positive role of SisArnE in DDR. Finally, transcriptomic analysis reveals that SisArnA represses a number of genes, implying that archaea apply the FHA/phospho-peptide recognition module for extensive transcriptional regulation.

**IMPORTANCE:**

Cellular adaption to diverse environmental stresses requires a signal sensor and transducer for cell survival. Protein phosphorylation and its recognition by forkhead-associated (FHA) domain proteins are widely used for signal transduction in eukaryotes. Although FHA proteins exist in archaea and bacteria, investigation of their functions, especially those in DNA damage response (DDR), is limited. Therefore, the evolution and functional conservation of FHA proteins in the three domains of life is still a mystery. Here, we find that an FHA protein from the hyperthermophilic Crenarchaeon *Saccharolobus islandicus* (SisArnA) represses the transcription of pili genes together with its phosphorylated partner SisArnB. SisArnA derepression facilitates DNA exchange and repair in the presence of DNA damage. The fact that more genes including a dozen of those involved in DDR are found to be regulated by SisArnA implies that the FHA/phosphorylation module may serve as an important signal transduction pathway for transcriptional regulation in archaeal DDR.

## INTRODUCTION

Cells in various environmental stresses yield adaptation to the ensure survival of living organisms. One of the most important adaption mechanisms is post-translational modification, in which phosphorylation is the widely spread in all three domains of life (1). Phosphorylation can be recognized by multiple types of protein domains for signal transduction (2). The Forkhead-associated (FHA) domain is one of the phospho-peptide recognizing domains, preferring to bind phosphorylated threonine (pThr) (3, 4). FHA domains are not conserved in sequence, but they share a similar 3-D structure. The core domain consists of 11 β-sheet linked by loops, forming a sandwich-like structure. Several invariant residues in those loops including serine (Ser), arginine (Arg), and asparagine (Asn) are responsible for pThr recognition (4, 5).

FHA domain was first identified as a conserved domain in a subset of forkhead-family transcription factors. This group of FHA domain proteins harbors DNA-binding domain (DBD) and is involved in transcriptional regulation (6, 7). In addition, eukaryotic FHA domain-containing proteins also play impocartant roles in various biological processes such as cell growth, DNA damage response (DDR), and cell cycle regulation (4, 8, 9). Notably, multiple proteins involved in DDR and DNA double-stranded break (DSB) repair harbor FHA domains, such as kinases Rad53, Mek1, CHK2, the subunits NBS1/Xrs2 of the MRN/MRX complexes, and E3 ligase RNF8 which mediates the histone H2A ubiquitination (4, 10). Therefore, those FHA proteins transfer DDR cascade signals through phospho-peptide recognition and conduct different activities, such as phosphorylation, dephosphorylation, or ubiquitination, via the domains fused with their FHAs. Due to the complexity of eukaryotic DDR, the issues such as where the FHA domain originated and how it evolved during evolution are far from being clear (10, 11).

Archaea of the order Sulfolobales, belonging to the TACK superpylum, serves as good model microbes for evolutionary studies on many processes including DNA replication, transcription, recombination, and cell division and cell cycle checkpoint control. They harbor eukaryotic-like DNA metabolic proteins, simplified cell division machinery, and eukaryotic-like cell cycle (12 - 14). The DDR in these model species was revealed to rely on an Orc1-2-centered transcriptional regulatory network (15). In this network, the transcription regulator Orc1-2 mediated the transcription of a number of genes for cell survival, including those that are involved in DNA exchange/repair (up-regulated) and cell cycle progress (down-regulated) (15, 16). However, Orc1-2 only binds to the promoters of certain up-regulated genes which contain a conserved motif for activation (15). The regulatory details of the Orc1-2-centered network are not fully clarified, and how the network is triggered is still mysteries. Our previous studies showed that the genome of *Saccharolobus* (formally *Sulfolobus*) *islandicus* REY15A encodes multiple eukaryotic-like kinases, and there exists a global phospho-proteomic change under ultraviolet (UV) irradiation (17, 18). FHA proteins are only conserved in the Crenarchaeota in archaea. Therefore, whether FHA proteins play a role in DDR in archaea is of scientific interest. Several studies on the FHA proteins in Sulfolobales showed that this protein was involved in the regulation of archaella synthesis and cell motility via interaction with the von Willebrand type A (vWA) domain-containing protein ArnB (19 - 22). Our previous study showed that *S. islandicus* FHA protein SisArnA is able to interact with SisArnB and another vWA domain protein SisvWA2. Both SisArnB and SisvWA2 contain multiple phosphorylation sites at C-terminal domains, and their phosphorylation levels are reduced after UV irradiation (18). Since we showed that SisvWA2 is also involved in the DDR regulatory network, this protein is renamed as SisArnE hereafter [ArnC and ArnD are two protein kinases that phosphorylate ArnA and/or ArnB in *S. acidocaldarius* (23)]. More importantly, it is interesting to know whether FHA proteins in Sulfolobales act as a general regulator in signal transduction for various stress responses. In addition, a study on archaeal FHA proteins could provide insight into the functional origin of FHA domain protein.

In this study, we performed genetic, biochemical, and transcriptomic analysis to investigate the roles of SisArnA in archaeal DDR signal transduction. We found that SisArnA deletion increases cell viability and cell aggregation in the presence of DNA damage. SisArnA inhibits the transcription of UV-induced pili genes (*ups*) via direct binding to the promoters of several *ups* genes together with SisArnB. The interactions of SisArnA with SisArnB and SisArnE were stimulated by phosphorylation of the vWA proteins *in vitro*. In addition, during DDR, the interaction between SisArnA and SisArnB was reduced resulting in the removal of the transcriptional inhibition, while the interaction of SisArnA and SisArnE was enhanced. Finally, our transcriptomic analysis indicates that SisArnA inhibits dozens of genes, implying that it may function as a global repressor in the cell.

## RESULTS

### The *SisarnA* deletion strain exhibits higher resistance to NQO compared to the wild-type strain

**Fig 1:**
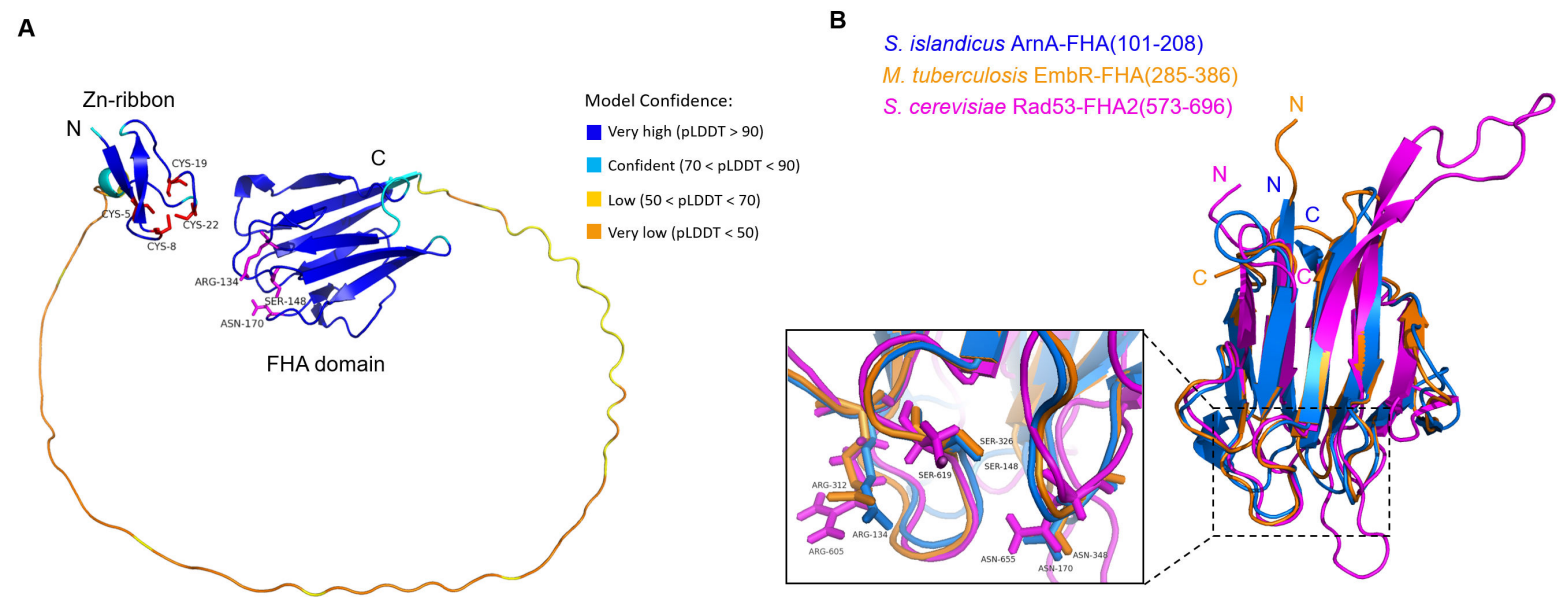
Structural analysis of the FHA domain-containing protein from *S. islandicus* (SisArnA, 208 aa). (**A**) Structural model of the whole SisArnA. The prediction was performed by the AlphaFold2 program (25), which produces a per-residue confident score (pLDDT, 0–100) on the local Distance Difference Test (lDDT-Cα) (25). The model confidence is shown in color, and the regions below 50 pLDDT may be unstructured in solution. Four cysteine residues (Cys-5, 8, 19, and 22) in the N-terminal Zn-ribbon domain are colored in red, while the three conserved residues (Arg-134, Ser-148, and Asn-170) for the recognition of phosphorylated threonine residue in the C-terminal FHA domain are colored in pink. (**B**) Superimposition of SisArnA (blue) with the FHA domains from *Mycobacterium tuberculosis* EmbR (orange) and *Saccharomyces cerevisiae* Rad53 (pink). The enlarged figure shows the loops containing three conserved pThr-binding residues.

FHA domain-containing proteins in eukaryotes play critical roles in signal transduction during DDR and DSB repair (4). Gene annotation indicates that SiRe_1010 of *S. islandicus* REY15A encodes an FHA-containing protein which we name as SisArnA (24). A 70-amino acids flexible loop links the FHA domain and a Zinc-ribbon domain, which are located at the C-terminal and N-terminal end, respectively (Fig. 1A). The flexible loop contains multiple QQ motif and is conserved in Sulfolobales. Structural modeling using Alphafold2 showed that the FHA domain of SisArnA was highly similar to those of bacterial and eukaryotic FHAs, with the former being a little closer both in the arrangement of loop/β-sheet and pThr binding residues (Fig. 1B).

**Fig 2:**
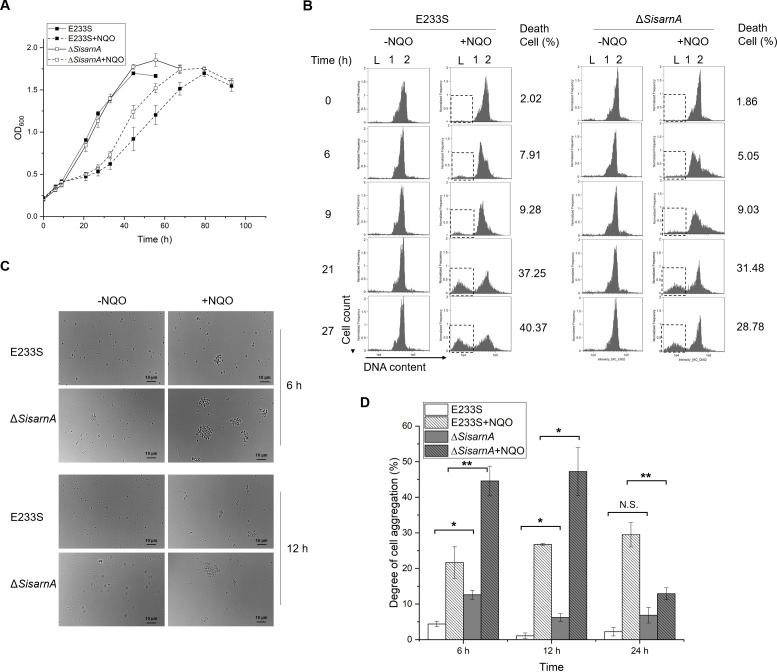
Comparison of the growth, flow cytometry profiles, and cell aggregation between Δ*SisarnA* and the wild-type strain E233S in the presence (+) and absence (−) of DNA damage agent 4-nitroquinoline 1-oxide (NQO). (**A**) Growth curves. The cultures were grown to OD_600_ = 0.2 before the DNA damage agent NQO (3 µM) was added. The values of OD_600_ were obtained from three independent cultures. Error bars indicate standard deviations. (**B**) Flow cytometry profiles. L, DNA-less cells; 1, cells containing one genome copy; 2, cells containing two genome copies. The ratios between the numbers of dead cells (boxed) and the total cell numbers at 6, 9, 21, and 27 h are indicated on the right in NQO-treated samples. (**C**) Cell aggregation analysis. Representative pictures are shown for the samples untreated or treated with NQO for 6 and 12 h. (**D**) Quantification of the results in (**C**). Aggregates containing more than three cells were counted. The values were calculated based on three independent experiments. One-tailed Student’s *t*-test was used for the statistical analysis. Significant: *P < 0.05, **P < 0.01, ***P < 0.001, N.S., not significant.

To understand whether SisArnA is also involved in DDR and/or DNA repair in Sulfolobales, the *SisarnA* gene was deleted by genome editing using the endogenous CRISPR-Cas-based system of *S. islandicus* (Fig. S1), and the phenotype of the deletion mutant was analyzed. Firstly, we determined the growth of Δ*SisarnA* in the presence and absence of DNA-damaging agent 4-nitroquinoline 1-oxide (NQO), which can cause UV-mimic DNA lesions (1). As shown in Fig. 2A, there was no growth difference between the wild-type E233S and Δ*SisarnA* under normal conditions, suggesting that the generation time of E233S did not change after *SisarnA* deletion. Interestingly, Δ*SisarnA* grew faster than the wild-type strain in the medium supplemented with NQO. Moreover, cell viability analysis revealed that Δ*SisarnA* had a higher survival ratio compared with E233S in the presence of NQO (40.91% ± 3.02% vs 31.72% ± 1.39%, Student *t*-test *P* < 0.05), indicating that Δ*SisarnA* is more resistant to the DNA damage agent than the wild type. To further confirm the function of SisArnA, we constructed a SisArnA overexpression strain (E233S/pSeSD-*N*-His-SisArnA) and tested its growth in the presence of NQO when the protein is highly induced in the medium containing arabinose. As expected, the SisArnA overexpression strain exhibited higher sensitivity to NQO, while it grew similar to the wild type when the strain was grown in the sucrose-containing medium (Fig. S2A and C). In addition, we also performed SisArnA complementation assay and showed that the complementation strain Δ*SisarnA*/pSeSD-His-SisArnA restored the NQO sensitivity compared with the deletion strain containing an empty vector (Δ*SisarnA*/pSeSD) (Fig. S2B and C). To know why the growth was affected, we performed flow cytometry analysis (Fig. 2B). It was shown that Δ*SisarnA* had less cells arrested in G1/S phase (1 copy of chromosome peak, 1C peak) than E233S at 6 and 9 h after NQO treatment. As time passed, the proportion of the DNA less cells (<1C, representing dead cells) in Δ*SisarnA* sample was less than that in E233S (31.5% vs 37.3% at 20 h, 28.8% vs 40.4% at 27 h). We find that Δ*SisarnA* also had slightly more cells with >2C peak than that for E233S in the presence and absence of NQO, which may contain some cell aggregates. Since no growth difference was observed between the wild type and Δ*SisarnA* in the absence of NQO, the aggregated cells in the sample of Δ*SisarnA* should be all alive. The result suggested that *SisarnA* deletion is beneficial for cell survival in the presence of the DNA damage agent.

One of the best-studied DDR response in Sulfolobales cells is that they are prone to aggregation in the presence of DNA lesions caused by UV irradiation or NQO, by which DNA exchange occurs between cells (26 - 28). Cell aggregation is mediated by UV-induced pili, the components of which are encoded by a cluster of *ups* genes (26, 27). To analyze the DDR response in Δ*SisarnA*, cell aggregation with or without NQO treatment was examined. We found that Δ*SisarnA* cells already aggregated at a higher ratio (11.7%) than E233S cells (4.4%) in the absence of NQO (Fig. 2C and D). After the cells are treated with NQO for 6 and 12 h, the cell aggregation in Δ*SisarnA* increased further than that in the wild type (44.5% vs 21.6% at 6 h, 47.5% vs 26.7% at 12 h) (Fig. 2C and D). However, at 24 h, the aggregation of Δ*SisarnA* was decreased to 13%, while that of the wild type was still around 30%. This indicated that the response of Δ*SisarnA* to DNA damage was faster and more efficient than that of the wild type.

### *Sisfha* deletion derepresses the transcription of the DDR genes

**Fig 3:**
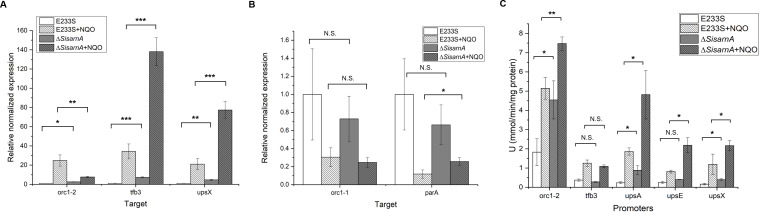
The expression of DDR genes is stimulated in Δ*SisarnA*. (**A**) RT-qPCR analysis of the expression of representative up-regulated DDR genes, *orc1-2*, *tfb3*, and *upsX*. The cells of E233S or Δ*SisarnA* were untreated or treated with NQO (3 µM) for 6 h, and total RNA was extracted and purified as described in the Materials and Methods. The comparative Ct value of tbp cDNA was used as a reference. The data were obtained from three independent experiments. (**B**) RT-qPCR analysis of the expression of the down-regulated DDR genes, *orc1-1* and *parA*. (**C**) Promoter activity analysis of the up-regulated DDR genes in E233S and Δ*SisarnA*. The strains carrying the plasmids containing the promoters (−200 to −1) of *orc1-2*, *tfb3*, or *upsX* were cultivated at OD_600_ = 0.2–0.3 and untreated or treated with 3-µM NQO for 6 h. The cells were collected and disrupted by sonication. Total cell extracts were subjected to ONPG assay (see the Materials and Methods). The values were obtained based on data from three independent experiments. Error bars indicate standard deviations. One-tailed Student’s *t*-test was used for statistical analysis. Significant: *P < 0.05, **P < 0.01, ***P < 0.001, N.S., not significant.

It was demonstrated that there exists an Orc1-2-centered DDR network in *Sulfolobus* and *Saccharalobus* through which dozens of genes are regulated, facilitating DNA repair (15, 26, 29). These consist of up-regulated genes such as *tfb3*, *ups*, and *ced* which are involved in DNA damage regulation and DNA exchange as well as down-regulated genes which are involved in DNA replication and cell division (15, 16). To test the effect of SisArnA on the transcription of DDR genes, RT-qPCR was employed to detect the transcription levels of *orc1-2*, *tfb3*, and *upsX* genes at the early log phase (OD_600_ = 0.2–0.3). Orc1-2 activates *tfb3* and *ups* which are also activated by Tfb3. Consistent with the growth and cell morphology of Δ*SisarnA*, the mRNA levels of all the three genes in Δ*SisarnA* were higher than those in E233S. After 6-h NQO treatment at OD_600_ = 0.2–0.3, the mRNA levels of *tfb3* and *upsX* further increased (Fig. 3A). However, the transcription of *orc1-2* was not higher than E233S in the presence of NQO. In addition, we also analyzed the transcriptions of two down-regulated genes in Orc1-2-centered DDR network, *orc1-1* and *parA*, which encode the proteins for DNA replication initiation and DNA segregation, respectively. But no difference was observed between E233S and Δ*SisarnA* (Fig. 3B). The results suggested that the phenotype of higher cell aggregation and more efficient response to DNA damage agent in Δ*SisarnA* was due to elevated transcription levels of *ups*. It may be also due to the higher transcription level of *tfb3*.

To confirm that SisArnA has a repressive role in the transcription of *orc1-2*, *tfb3*, and *ups* genes, we conducted a promoter activity assay based on the β-glucosidase activity system. The promoter regions of *orc1-2*, *tfb3*, and *ups* (*upsA, upsE*, and *upsX*) (200 bp upstream of their start codons) were used for the analysis. E233S and Δ*SisarnA* harboring pSe-Porc1-2/tfb3/upsA/upsE/upsX-SsolacS at the early log phase were treated or mock treated with NQO. The cells were collected at 6 h after treatment, and the β-galactosidase activity of cell extracts was measured. As shown in Fig. 3C, the promoter activities of *orc1-2, upsA*, *upsE,* and *upsX* in Δ*SisarnA* increased compared with those in E233S in both the presence and absence of NQO, while the promoter activity of *tfb3* did not change significantly in Δ*SisarnA*. These results reinforce that SisArnA is a transcriptional repressor of the DDR genes, with the *ups* genes being the most strongly repressed. However, the results from the promoter activity assay for *orc1-2* and *tfb3* were not consistent with their qPCR data. SisArnA inhibited orc1-2 promoter activity but did not affect its mRNA in the presence of NQO implying that elevated downstream regulation of Orc1-2 in Δ*SisarnA* might be high enough for the DNA exchange and repair, and there may be a complicated regulation network through which the RNA level of Orc1-2 is reduced for turning off the DDR. The result that SisArnA inhibited *tfb3* transcription but not its promoter region may suggest that the 200 bp promoter of tfb3 was not a main region targeted by SisArnA.

### Genetic interaction analysis of *SisarnA, SisarnB*, and *SisarnE*

**Fig 4:**
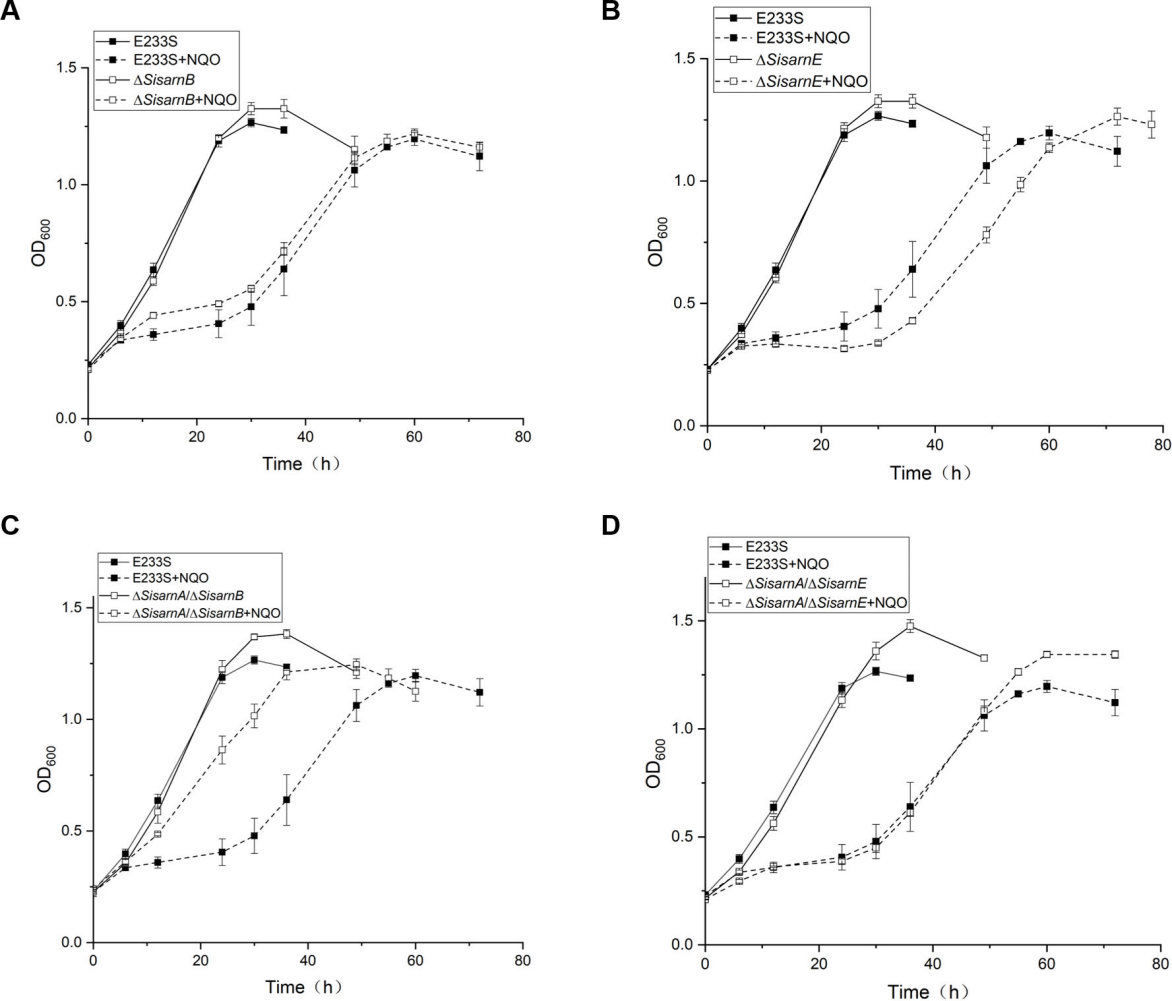
Growth curves of Δ*SisarnB* (**A**), Δ*SisarnE* (**B**), Δ*SisarnA/*Δ*SisarnB* (**C**), and Δ*SisarnA*/Δ*SisarnE* (**D**) in the absence or presence of NQO. The procedure is the same as described in Fig. 2. The experiments were repeated for three times. The data points for the E233S growth curves are the same as in panels A–D. Error bars indicate standard deviations.

It was reported that *Sac*ArnA and *Sac*ArnB (homologs of SisArnA and SisArnB, respectively, in *S. acidocaldarius*) interacted with each other and worked together in the regulation of *archaellum* (*arl*) genes which encodes the proteins for cell mobility (19 - 21). As interactions of SisArnA with both SisArnB and SisArnE have been detected in our previous study (18) and SisArnA is found to be involved in DDR, it is reasonable to ask whether SisArnB and SisArnE participate in DDR together with SisArnA. To answer this question, we constructed additional strains Δ*SisarnB*, Δ*SisarnE*, Δ*SisarnA*Δ*SisarnB*, and Δ*SisarnA*Δ*SisarnE* (Fig. S1). The sensitivities of these strains to NQO were analyzed with growth curves (Fig. 4). We found that Δ*SisarnB* exhibited slightly higher resistance to NQO than E233S within 30 h after treatment, while Δ*SisarnE* was more sensitive to NQO than E233S. In addition, the growth of Δ*SisarnA*Δ*SisarnB* was better than that of the single mutants Δ*SisarnA* and Δ*SisarnB* in the presence of NQO, suggesting that SisArnA and SisArnB may work together for the regulation of DDR genes. SisArnA probably plays a major role in this process although other proteins or pathways may also be involved. On the other hand, the sensitivity of Δ*SisarnA*Δ*SisarnE* was similar to that of the wild-type strain. Considering that Δ*SisarnA* and Δ*SisarnE* exhibited opposite responses to NQO and the proteins interact with each other, we speculate that SisArnE functioned as an activator during DDR in association with SisArnA.

**Fig 5:**
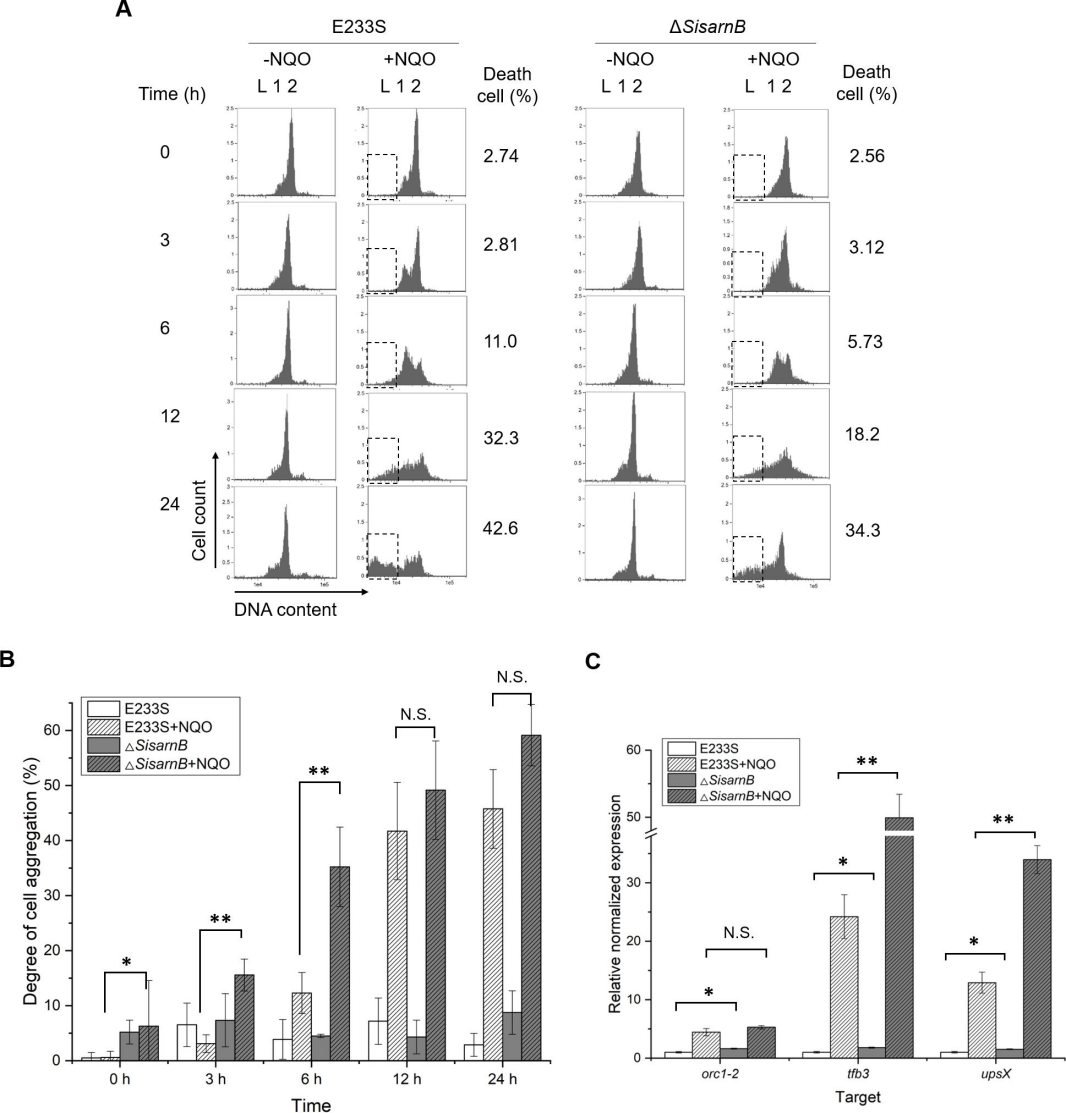
Comparison of the flow cytometry profiles, cell aggregation, and the transcription of the DDR genes between Δ*SisarnB* and the wild-type strain E233S in the presence (+) and absence (−) of NQO. (**A**) Flow cytometry profiles. L, DNA-less cells; 1, cells containing one genome copy; 2, cells containing two genome copies. The ratios between the numbers of dead cells (boxed) and the total cell numbers at 0, 3, 6, 12, and 24 h are indicated on the right. (**B**) Cell aggregation analysis. The aggregates containing more than three cells with or without NQO treatment at 0, 3, 6, 12, and 24 h were quantified. The values were calculated based on three independent experiments. (**C**) RT-qPCR analysis of the transcripts of the DDR genes. E233S or Δ*SisarnB* cells were untreated or treated with NQO for 6 h, and their total RNA was extracted for RT-qPCR with corresponding qPCR primers of *orc1-2*, *tfb3*, and *upsX*. The data were obtained from three independent experiments. Error bars indicate standard deviations. The comparative Ct value of tbp cDNA was used as a reference. One-tailed Student’s *t*-test was used for statistical analysis. Significant: *P < 0.05, **P < 0.01, ***P < 0.001, N.S., not significant.

To further confirm the phenotype of Δ*SisarnB*, the DNA content, cell aggregation, and transcription levels of the DDR genes were also analyzed. As expected, the ratios of DNA less cells (<1C) in Δ*SisarnB* were lower than those of wild type in the presence of NQO (Fig. 5A). We found that the proportion of cells in aggregation in Δ*SisarnB* increased at 3 and 6 h after NQO treatment compared with those of E233S although it did not change apparently in the absence of NQO (Fig. 5B). RT-qPCR analysis on the transcription of *orc1-2*, *tfb3*, and *upsX* genes revealed that the mRNA levels of *tfb3* and *upsX* genes also increased compared with those in E233S, especially after NQO treatment for 6 h (Fig. 5C). These results indicated that SisArnB interacted with SisArnA and worked as an inhibitor complex during *S. islandicus* DDR.

We also analyzed the phenotype of Δ*SisarnE*. Consistent with the growth curve, Δ*SisarnE* had more DNA less cells than E233S (Fig. S4A). However, we found that the ratios of cells in aggregation in Δ*SisarnE* were higher than those in the wild type after NQO treatment at 6 and 12 h (Fig. S4B). This is consistent with the RT-qPCR result that the mRNA levels of *tfb3* and *upsX* genes were further increased in Δ*SisarnE* compared with those in E233S in the presence of NQO (Fig. S4C). Notably, under normal growth conditions, the transcriptions of *tfb3* and *upsX* were not significantly different between E233S and Δ*SisarnE* (Fig. S4C), suggesting that SisArnE did not repress the expression of these genes *in vivo*. The higher cell aggregation ratio and transcriptional levels of *tfb3* and *upsX* genes in Δ*SisarnE* may be due to more lesions induced by NQO in this strain than E233S, leading to higher sensitivity to NQO.

### The interaction between SisArnA and SisArnB was reduced but that with SisArnE was enhanced after DDR

**Fig 6:**
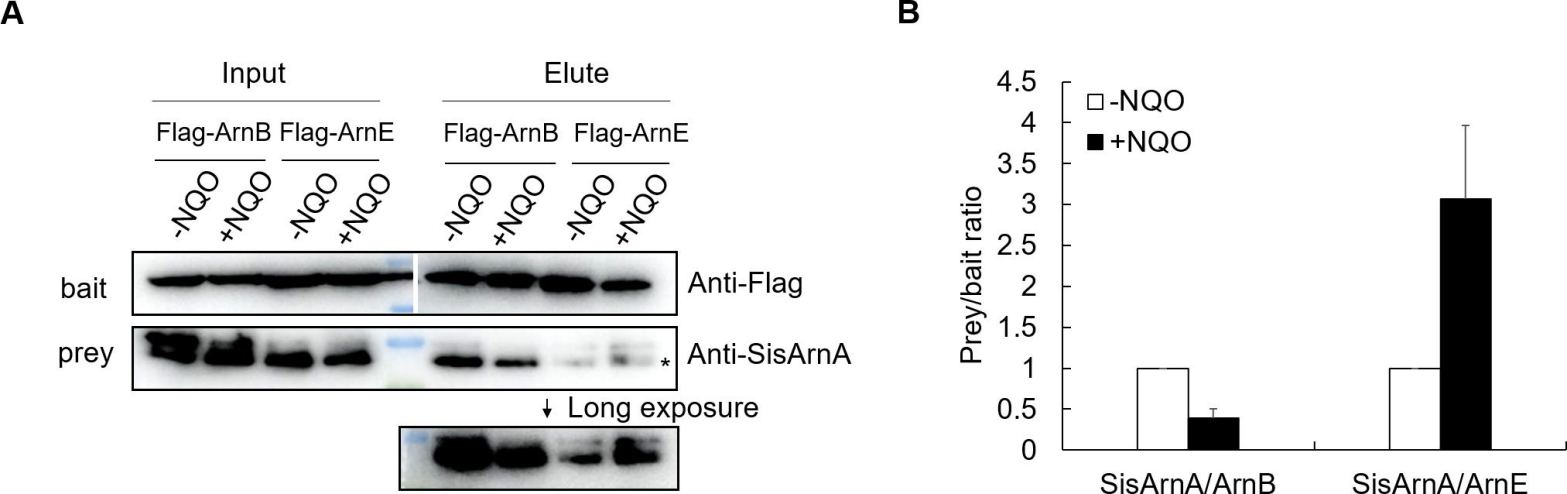
Pull-down analysis of the association between SisArnA and SisArnB or SisArnE in the presence and absence of NQO *in vivo*. (**A**) Western blotting analysis of SisArnA pulled by FLAG-tagged SisArnB or SisArnE. The complementation strains Δ*SisarnB*/pSeSD-Flag-ArnB and Δ*SisarnE*/pSeSD-Flag-ArnE were cultivated to OD_600_ = 0.2–0.3 in the arabinose-containing medium. The cells were treated with 3 µM NQO for 6 h and collected. Flag-ArnB and Flag-ArnE were precipitated with anti-Flag-coated magnetic beads. The input and elute samples were analyzed by Western blotting with anti-Flag and anti-SisArnA antibodies (see the Materials and Methods for detail). The experiments were performed for three times independently, and the representative gels are shown. The figure is an overlay between images taken after exposure and under light. The asterisk indicates the position of SisArnA whose signals were used for quantification. (**B**) Quantification of the results in **A**. SisArnA/ArnB (or ArnE) ratio without NQO treatment was set to 1.

Our previous proteomic study revealed that the phosphorylation levels of SisArnB and SisArnE were reduced after UV treatment for 30 min (18). Therefore, we speculated that the interactions of SisArnA with SisArnB and SisArnE may be inhibited in the presence of DNA damage *in vivo*. To determine this, we cultivated the complementation strains Δ*SisarnB*/pSeSD-Flag-ArnB and Δ*SisarnE*/pSeSD-Flag-ArnE and purified Flag-tagged SisArnB and SisArnE from their corresponding strains treated or mock-treated with NQO for 6 h. The interacted SisArnA pulled down by SisArnB or SisArnE was detected by Western blot with anti-SisArnA antibody. We found that the interaction of SisArnA-ArnB was stronger (about nine times) than that of SisArnA-ArnE under normal growth conditions, indicating that SisArnB was a main interactor of SisArnA *in vivo* (Fig. 6A and B). Strikingly, after NQO treatment, the amount of SisArnA pulled by SisArnB was reduced to 40% of the untreated sample, while that of SisArnA interacted with SisArnE increased to threefold of the untreated sample (Fig. 6A and B). To confirm that phosphorylation would affect the interactions of SisArnA with SisArnB and SisArnE, we purified these proteins from *Escherichia coli* and performed *in vitro* pull-down assay in the absence or presence of the kinase ePK1 (SiRe_2056) (18) (Fig. S5A). The result showed that phosphorylation of SisArnB and SisArnE stimulated their interactions with SisArnA, respectively (Fig. S5B). Since the phosphorylation levels of SisArnB and SisArnE were reduced at 30 min after UV treatment in our previous study (18), we could not exclude that their phosphorylation would be changed at the later stage of DDR, resulting in different interaction capabilities with SisArnA. The different responses of the interactions of SisArnA-ArnB and SisArnA-ArnE were in agreement with the contrasting phenotypes of Δ*SisarnB* and Δ*SisarnE*, further suggesting that SisArnB was a repressor, whereas SisArnE was a positive regulator after DDR.

### SisArnA in combination with SisArnB exhibited higher DNA-binding activity toward the promoter DNA

To reveal how SisArnA and SisArnB inhibit the transcription of *orc1-2*, *tfb3*, and *ups* genes, electrophoretic mobility shift assay (EMSA) was performed to detect the DNA-binding activity of SisArnA, SisArnB, and SisArnE on the promoters of these DDR genes. His-SisArnB, His-SisArnE, and untagged SisArnA were heterologously expressed and purified from *E. coli* (Fig. S5A). FAM-labeled promoter DNA of *orc1-2*, *tfb3*, and *upsX* was used as substrates. All SisArnA, SisArnB, and SisArnE were unable to bind any promoter individually (Fig. S6A). Interestingly, DNA binding was observed in the presence of both SisArnA and SisArnB, or SisArnA and SisArnE, although the activity was low (Fig. S6A).

**Fig 7:**
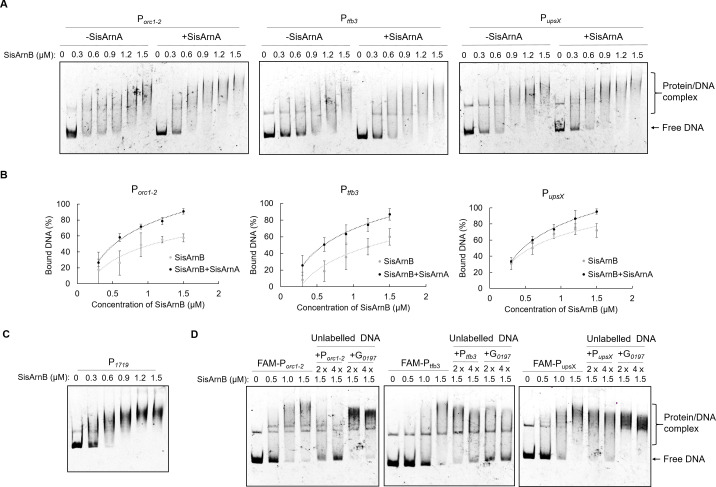
DNA-binding activities of SisArnA and SisArnB on the promoters of representative DDR genes. SisArnA and SisArnB were purified from their corresponding overexpression strains of *S. islandicus*. Different concentrations (μM) of SisArnB, either alone or together with 2 µM SisArnA, were incubated in a reaction mixture containing FAM-labeled promoter DNA fragment (200 bp) of *orc1-2*, *tfb3*, or *upsX*. The samples were analyzed in 8% native PAGE gel. The gels were scanned using Amersham ImageQuant 800. (**A**) The gel profiles of EMSA on the promoters of *orc1-2*, *tfb3*, and *upsX*. (**B**) Quantification of the results in **A**. The bound DNA was quantified. At least three independent experiments were performed, and the representative gels are shown. Error bars indicate standard deviations. (**C**) The SisArnB sample bound an unspecific promoter. The FAM-labeled promoter of *SiRe_1719* (encoding the histidyl-tRNA synthetase) was used for the EMSA assay. (**D**) DNA competition assay. Unlabeled promoter DNA fragments of *orc1-2*, *tfb3*, *upsX* or the gene fragment of *SiRe_0197* was added into the reaction mixtures at the concentrations of two- or fourfold of labeled DNA for the competition.

Next, we detected the activities of the proteins purified from *S. islandicus*. His-SisArnA, Flag-SisArnB, and Flag-SisArnE were purified from their corresponding overexpression strains, individually (Fig. S5A). Surprisingly, the EMSA assay revealed that the SisArnB sample exhibited DNA-binding activity on all the three promoters (Fig. 7A), different from the protein from *E. coli*. Because SisArnB interacted with SisArnA more strongly than that of SisArnE *in vivo*, we have detected that a few amount of SisArnA was co-purified together with Flag-SisArnB from *S. islandicus* (Fig. 6A). Therefore, we speculated that both Flag-SisArnB and SisArnA in the sample contributed to the DNA-binding activity. To confirm this, Flag-SisArnB was purified from an overexpression strain with the *arnA* deletion background (Δ*arnA*/pSeSD-Flag-ArnB). As expected, this sample without ArnA was unable to bind the three promoters at the same concentrations (Fig. S6B; Fig. 7A and B). Then, all three promoters were examined, and the result showed that the SisArnB sample had higher affinity to the *upsX* promoter (P*_upsX_*) compared to the other two promoters (Fig. 7A and B). Although SisArnA did not exhibit DNA-binding activity toward different promoters, the addition of SisArnB in the reaction with the SisArnA sample further enhanced the DNA-binding capability (Fig. 7A and B), consistent with the EMSA result using proteins purified from *E. coli*. To detect the specificity of DNA-binding ability by SisArnA-SisArnB, the FAM-labeled promoter of *SiRe_1719* was used for the DNA-binding assay. *SiRe_1719* encodes the histidyl-tRNA synthetase, and its transcription was not affected by either NQO treatment or *SisarnA* deletion [(15) and Table S3]. We found that the promoter P*_SiRe_1719_* could also be bound by SisArnA and SisArnB comparable with the promoters of *orc1-2*, *tfb3*, and *upsX* (Fig. 7C). In addition, a DNA competition assay was performed using unlabeled promoters of *orc1-2*, *tfb3*, *upsX*, and a gene fragment (*sire_0197*). We found that the bound FAM-labeled P*_orc1-2_*, P*_tfb3_*, and P*_upsX_* can be replaced with their corresponding unlabeled promoters (P*_orc1-2_*, P*_tfb3_*, or P*_upsX_*), while the gene fragment of *SiRe_0197* was unable to compete with P*_orc1-2_* and P*_upsX_* and only partially compete with P*_tfb3_* in the EMSA assay (Fig. 7D), suggesting that SisArnA-SisArnB exhibited higher DNA-binding activity toward promoter sequences. The lower binding activity of SisArnA-SisArnB on P*_tfb3_* was consistent with the promoter activity assay (Fig. 3C) showing that the selected 200 bp promoter region of *tfb3* may not be the main target of SisArnA-SisArnB. Unexpectedly, we found that the SisArnE sample from *S. islandicus* degraded these DNA substrates, and the degradation was inhibited by the addition of EDTA (Fig. S6C). Our results indicated that SisArnA-SisArnB inhibited the transcription of *orc1-2* and *ups* genes by directly binding to their promoters.

We found that the DNA-binding activity of SisArnA-ArnB purified from *S. islandicus* was stronger than those from *E. coli*. Given the fact that SisArnB was phosphorylated at multiple residues in its host cell, the higher DNA-binding activity should be due to the stronger interaction of SisArnA-ArnB than the proteins purified from *E. coli*, which was not phosphorylated or in low phosphorylation (18). As expected, Western blot analysis demonstrated that the phosphorylation level of the SisArnB sample purified from *S. islandicus* was much higher than SisArnB purified from *E. coli*, in which the phosphorylation can be removed by the phosphatase PP2A (Fig. S6D). To further confirm this, SisArnB, purified from *E. coli*, was phosphorylated by ePK1 *in vitro* for EMSA. We found that the phosphorylated SisArnB exhibited slightly higher DNA-binding activity in the presence of SisArnA, compared to its un-phosphorylated form (Fig. S6E).

### Transcriptomic analysis of *SisarnA* deletion in the presence or absence of NQO

To further understand whether there are other genes or pathways regulated by SisArnA, we conducted transcriptomic analysis of *SisarnA* deletion mutant treated with NQO or without treatment compared with those of the wild-type E233S. The culture samples were taken at 6 h after treatment, and total RNA was isolated for reverse transcription and sequencing. It was shown that 72 genes were up-regulated and eight genes were down-regulated after *SisarnA* deletion. Consistent with our RT-qPCR results, the transcripts of *tfb3* and the *ups* genes significantly increased (*tfb3* 2.23-fold, *upsA* 3.46-fold, *upsB* 2.66-fold, and *upsE* 3.39-fold) (Table 1). However, no transcriptional change was observed for *orc1-2*. We found that the transcriptional levels of several *arl* genes, which encode for *Sulfolobus* archaella components, were also slightly increased (*SiRe_0121* 2.95-fold, *SiRe_0122* 1.87-fold, and *SiRe_0124* 1.88-fold) (Table S3) in agreement with the previous study that *S. acidocaldarius* FHA protein ArnA inhibited the biosynthesis of *Sulfolobus* archaella (19). Strikingly, a dozen of putative DDR genes highly induced in the presence of NQO was also up-regulated in the untreated Δ*SisarnA* (Table 1). In addition, multiple up-regulated genes were significantly enriched in several metabolic pathways, including oxidative phosphorylation, TCA cycle, butanoate and pyruvate metabolism, and glycolysis/gluconeogenesis, in Δ*SisarnA* (Fig. S7). Moreover, there were three gene clusters transcriptionally increased, including those involved in terpenoid backbone biosynthesis (SiRe_1459–1462) and sulfur metabolism (SiRe_2307–2309 and SiRe_2310–2313) (Table S3). SiRe_1459 in the cluster SiRe_1459–1462 was annotated as a 3-hydroxy-3-methylglutaryl coenzyme A (HMG-CoA) reductase which was supposed to be involved in terpenoid backbone biosynthesis. Terpenoid compounds, such as sterols, carotenoids, or the prenyl groups of various proteins, are synthesized via the mevalonate pathway (30). A rate-limiting step of this pathway is the conversion of HMG-CoA to mevalonic acid catalyzed by the HMG-CoA reductase (31). It was reported that the activity of HMG-CoA may affect various biological processes including cellular adaption to environmental changes (32, 33). Another two clusters, SiRe_2307–2309 and SiRe_2310–2313, encode proteins participating in sulfur metabolism. The sulfur oxidation and reduction are critical metabolic pathways for this model strain living in the environments containing sulfur (34, 35). Therefore, all these genes up-regulated may stimulate cell growth under certain stress.

**TABLE 1:** Selected DDR genes significantly changed in Δ*SisarnA* or NQO-treated strains^*a*^

Process	Gene ID^*b*^	Protein	Δ*arnA*/E233S	E233S_NQO/E233S	Δ*arnA*_NQO/Δ*arnA*	Δ*arnA*_NQO/E233S_NQO
DNA replication and transcription	SiRe_1231	Orc1-2	1.470708	3.257708	3.771665	1.716234
	**SiRe_1717**	Tfb3	2.233172	25.09828	28.75049	2.573739
	**SiRe_0614**	Dpo2_N_	1.988684	21.3448	14.84651	1.394922
	SiRe_0615	Dpo2_C_	1.862119	25.64527	18.38993	1.34491
	SiRe_0616	Hypothetical protein	1.82367	22.26642	15.44077	1.274619
DNA transfer	**SiRe_1316**	CedA1	2.276737	24.21235	21.35615	2.023409
	SiRe_1317	CedA	1.70288	22.85683	20.41567	1.530797
	SiRe_1878	UpsX	1.035141	14.74947	19.64543	1.38625
	**SiRe_1879**	UpsE	3.391792	39.85754	29.28945	2.508557
	SiRe_1880	UpsF	1.862119	25.64527	18.38993	1.34491
	**SiRe_1881**	UpsA	3.454051	47.23674	30.53604	2.244086
	**SiRe_1882**	UpsB	2.653082	31.28831	25.19258	2.150315
Oxidative stress and homologous recombination	**SiRe_0453**	Dps	2.460187	2.512386	2.346385	2.316452
	SiRe_0454	Rieske (2Fe-2S) protein	1.913858	2.257665	2.275646	1.943567
	SiRe_0061	NurA	1.078934	2.165202	3.103753	1.558742
	SiRe_0062	Rad50	0.867557	2.081714	3.773653	1.584017
	SiRe_0063	Mre11	0.991877	1.940167	3.839474	1.976835
	SiRe_0064	HerA	1.050394	1.987542	3.792621	2.019244
Other DDR-related proteins	**SiRe_0670**	Hypothetical protein	10.60699	69.14417	27.2711	4.213045
	**SiRe_0137**	Hypothetical protein	5.815725	56.12965	37.42254	3.911249
	**SiRe_2100**	Hypothetical protein	5.242094	42.25696	28.73379	3.599604
	**SiRe_0187**	Hypothetical protein	2.992531	31.64887	27.81672	2.644262
	**SiRe_1957**	Flagellar hook-basal body protein	2.985278	33.06059	30.48513	2.770726
	**SiRe_0269**	Hypothetical protein	2.620876	30.74186	27.96223	2.397798
	**SiRe_0589**	Hypothetical protein	2.615577	15.58869	16.44795	2.779191
	**SiRe_0020**	Hypothetical protein	2.427957	27.19279	24.40636	2.189312
	**SiRe_0115**	Hypothetical protein	2.420355	4.090435	2.615646	1.56073
DNA replication	SiRe_1740	Orc1-1	0.950516	0.372111	0.433747	1.117161
Chromosome segregation and cell division	SiRe_1172	Hypothetical protein	1.458071	0.472708	0.468007	1.455067
	SiRe_1173	CdvA	1.291688	0.276785	0.488724	2.29946
	SiRe_1174	ESCRT-III	1.193696	0.308186	0.445789	1.740458
	SiRe_1175	Vps4	1.273353	0.375153	0.41521	1.420794
	SiRe_1200	ESCRT-III-2	1.651032	0.362361	0.367019	1.686931
	SiRe_1388	ESCRT-III-3	0.90601	0.352731	0.442867	1.143165
	SiRe_1550	ESCRT-III-1	1.343703	0.399317	0.370995	1.259446
	SiRe_1961	SegB	1.612691	0.539648	0.384145	1.157039
	SiRe_1962	SegA	1.458844	0.344242	0.360059	1.538002

^*a*^ The mRNA abundant ratios are calculated based on FPKM values from three independent data.

^*b*^ The gene IDs in bold are those up-regulated more than twofold both after SisarnA deletion and NQO treatment.

**Fig 8:**
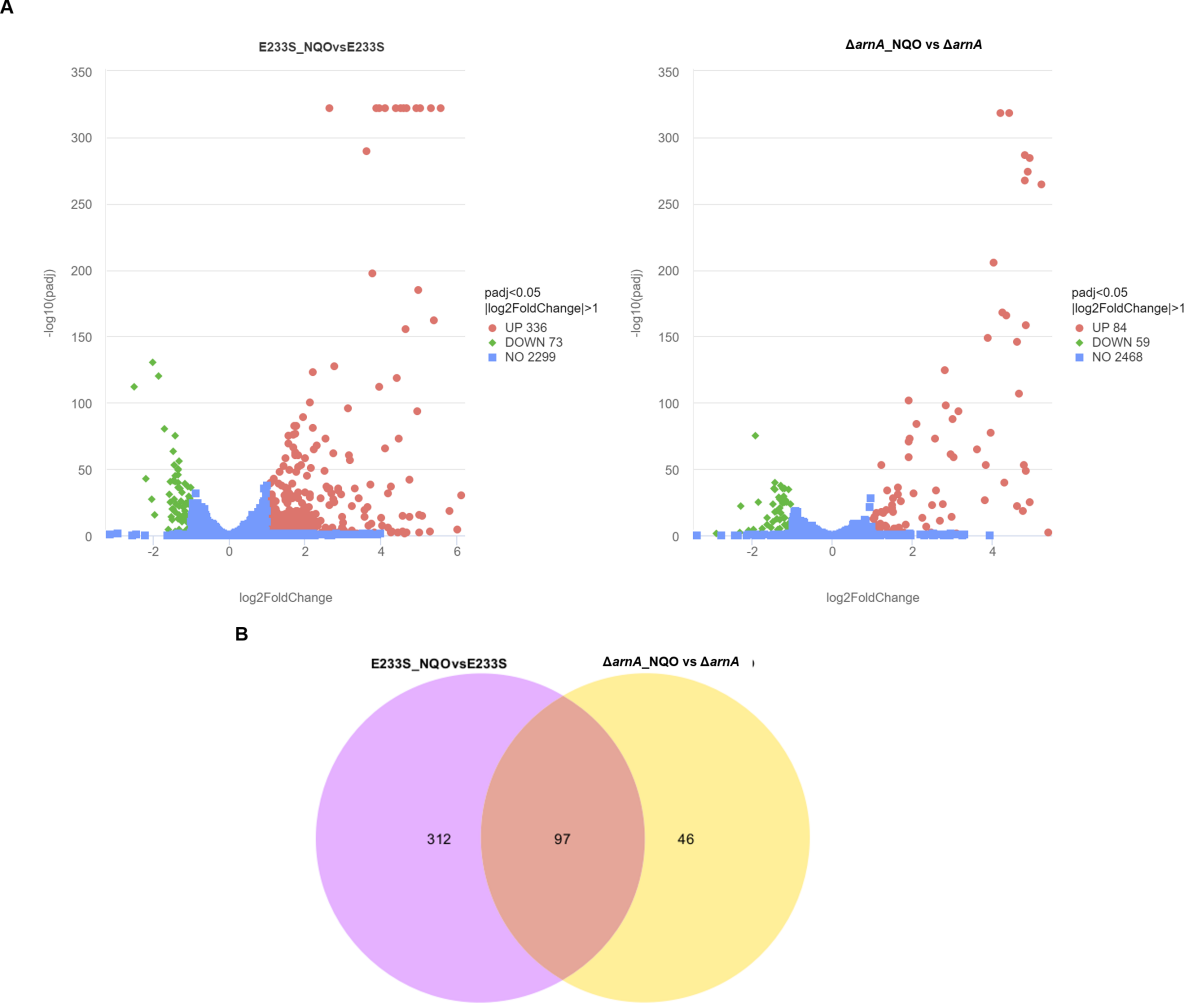
Transcriptomic analysis of E233S and Δ*SisarnA* after NQO treatment. (**A**) Volcano plot of differentially expressed genes in NQO-treated E233S and Δ*SisarnA* compared to their corresponding untreated strains. *X*-axis, fold change in gene expression. *Y*-axis, the significance of fold change. Genes exhibiting more than twofold (i.e., log_2_ fold change > 1) up- and down-regulated with significance (*P*_adj_ < 0.05) are highlighted in red and green, respectively, whereas those with less than twofold change in differential gene expression or with no significance are shown in blue. Three independent cultures of each strain were applied for the transcriptomic sequencing and statistical analysis. (**B**) Venn cluster of differentially expressed genes in E233S_NQO vs E233S and Δ*SisarnA*_NQO vs Δ*SisarnA*.

To analyze the putative effect of SisArnA on the Orc1-2-dependent DDR network, we compared the transcriptomic changes of E233S_NQO vs E233S with those of Δ*SisarnA*_NQO vs Δ*SisarnA*. As previously reported, NQO treatment triggered the Orc1-2-centered DDR network which induced *orc1-2, tfb3, ups, ced, dpo2, herA-mre11-rad50-nurA* operon for DNA exchange and repair and inhibited those genes involved in DNA replication, chromosome segregation, and cell division (Table 1). Nearly, all of these genes and those with unknown functions were changed in the same way in Δ*SisarnA*_NQO vs Δ*SisarnA* (Table 1), indicating that the major DDR was not affected by *SisarnA* deletion. In addition, the transcript levels of *tfb3* and *ups* genes were further increased in Δ*SisarnA*_NQO vs Δ*SisarnA* compared to those in E233S_NQO vs E233S (Table 1), consistent with our RT-qPCR results (Fig. 3A). However, a major part (about three-fourths) of other NQO responsive genes in E233S did not change significantly in Δ*SisarnA* after NQO treatment (336 up/73 down in E233S_NQO vs E233S compared to 84 up/59 down in Δ*SisarnA*_NQO vs Δ*SisarnA*) (Fig. 8; Table S3). Since the main DDR in the fundamental processes was unaffected and a number of the DDR genes were transcriptionally increased in Δ*SisarnA* in the absence of DNA damage, we speculated that since the response and recovery of Δ*SisarnA* to DNA damage was faster than the wild type, it was not necessary to further change the transcript levels of other genes such as those involved in cellular metabolism for the DDR.

## DISCUSSION

FHA domain is a specific module for phospho-Thr recognition which is found in many eukaryotes. The signal transduction roles of FHA domain proteins were extensively studied in eukaryotes, especially those involved in DDR for signal transduction and enzymatic activity (4). In addition, there is a family of transcription factors, containing both FHA and DBD domains, participating in multiple pathways in addition to transcription regulation. For example, the well-studied FHA proteins Fkh1 (forkhead homolog 1) and Fkh2 of *Saccharomyces cerevisiae* regulate the cell cycle by targeting various genes in different cell cycle phases (36, 37). Moreover, Fkh1 and Fkh2 also play a transcription-independent role in regulating DNA replication origin activation and yeast mating-type switch, via interaction with multiple proteins mediated by its FHA domain (38 - 40). Therefore, Fkh proteins participate in multiple pathways through interactions with target DNA and various proteins via their DNA binding and FHA domains. The bacterial FHA domain is fused with diverse domains and only widely distributed in three main groups: the mycobacteria and their relatives, the cyanobacteria, and the Gram-negative proteobacteria together with the Chlamydias (41). The FHA proteins in bacteria also exhibited diverse functions, including glutamate and lipid production, regulation of cell shape, type III and Type VI secretion, sporulation, and host-bacterium interactions. Nearly, all studies showed that FHA proteins regulate the activities of target proteins via protein-protein interaction (42 - 45). We found that FHA proteins are only conserved in Crenarchaeota and occasionally identified in other phyla in archaea. As previously reported, the FHA domain might originate in eukaryotes after the separation of archaeal and eukaryotic lineages and move to certain prokaryotes by horizontal gene transfer (2). And, the functions of FHA domains evolved diversely together with their fused domains in a late stage of evolution.

Here, we report that the FHA protein from *S. islandicus* (SisArnA) regulated gene transcription and interact with several proteins. Interestingly, our study revealed that SisArnA worked as a repressor for a number of DDR genes together with its partner SisArnB. It was revealed that SisArnB was phosphorylated at multiple residues *in vivo* (18), which would mediate the interaction with SisArnA. Consistently, we found that complementation of wild-type SisArnA, but not pThr-binding-deficient mutant R134A/S148A, inhibited cell viability in the presence of NQO (Fig. S2B and C). EMSA analysis revealed that both proteins in combination display DNA binding activity, indicating that the interaction between SisArnA and SisArnB mediated by phosphorylation is essential for the transcriptional inhibition of the DDR genes. In addition, SisArnA harbors a Zn-ribbon domain containing four cysteines (Cys) at its N-terminus (Fig. 1). This domain often functions as a nucleic acid-binding module (46). However, we found that DNA-binding activity of SisArnA was undetectable (Fig. S7A), indicating that the Zn-ribbon itself could not bind DNA. This is different from the FHA domain-containing eukaryotic transcription factors which can directly bind DNA via its DBD for their roles in replication initiation and transcription regulation (39). The function of Zn-ribbon domain in SisArnA needs further investigation. It is also interesting to investigate how SisArnA and SisArnB in combination exhibited DNA-binding activity, especially toward promoter sequences.

In addition to SisArnB, another vWA domain protein SisvWA2, which we designate as SisArnE in this study, was also one of the interactors of SisArnA *in vivo*. We found that SisArnA-SisArnB interaction was stronger than that of SisArnA-SisArnE interaction under normal growth conditions (Fig. 6). However, the interaction between the SisArnA and SisArnE homologs in *S. acidocaldarius* was not detectable (19), implying a species-specific model, or the interaction was too weak to be detected. Our genetic work revealed that, in contrast to SisArnB, SisArnE acts as a positive regulator for genes involved in DDR since its deletion reduced cell viability in the presence of NQO. Δ*SisarnA* grew better, while Δ*SisarnA/SisarnE* exhibited similar growth compared with that of the wild-type strain after NQO treatment. *In vivo* pull-down showed that SisArnA-SisArnB interaction was reduced, while SisArnA-SisArnE interaction was enhanced in the presence of DDR (Fig. 6), indicating that SisArnA and SisArnE might be involved in a same process after DDR occurred. One potential mechanism was that SisArnE interacted strongly with SisArnA and released the repression of SisArnA-SisArnB on some DDR genes in the presence of DNA damage. However, we cannot exclude that SisArnA-SisArnB and SisArnA-SisArnE may function on different groups of genes. Thus far, we did not know whether SisArnA-SisArnE could bind certain DDR gene promoters because the DNA substrates were degraded in the EMSA assay with the SisArnE sample purified from *S. islandicus*. Since SisArnE purified from *E. coli* did not degrade DNA and the vWA domain is a universal scaffold for PPI (47), the DNA degradation activity of the SisArnE sample from *S. islandicus* might be due to certain SisArnE partner *in vivo*. The specific function of SisArnE in DDR needs further investigation.

The transcriptomic analysis of *SisarnA* deletion mutants revealed that a number of genes, including many DDR genes, were up-regulated, indicating that SisArnA was a global inhibitor in *S. islandicus*. Although most of these genes were up-regulated less than sixfold in Δ*SisarnA*, the increased levels of these DDR genes might deal with DNA damage and regulate cell cycle more quickly, resulting in increased cell viability, as reported for the phenotype of Orc1-2 overexpression strain (15). According to our data, the Orc1-2-centered DDR network was not affected in Δ*SisarnA* after NQO treatment. Moreover, in the previous report, the transcription level of *SisarnA* did not change apparently with either NQO treatment or *orc1-2* deletion (15). These imply that phosphorylation-dependent regulation mediated by SisArnA might be in parallel with the Orc1-2-centered transcriptional regulation. Furthermore, we also analyzed the phenotypes (cell growth, cell aggregation, and transcription of the DDR genes) of Δ*SisarnA* with methyl methanesulfonate, which resulted in similar phenotypes with those in the presence of NQO (Fig. S8). On the other hand, although we did not have cell mobility data on Δ*SisarnA*, our transcriptomic result is in agreement with the previous study showing that *S. acildocaldarius* FHA protein inhibited the transcription of *arl* genes and regulated cell mobility during starvation, which is totally different environmental conditions from the ups pili (19, 20). It was reported that deletion of the genes encoding the adhesive pilus resulted in induction of the archaellum operon in *S. acidocaldarius* (48), implying that there is a complex regulatory network for different archaeal surface structures in which protein phosphorylation may be involved. Together, these indicate that archaeal FHA proteins might function in the regulation of various pili responding to various cellular stresses.

**Fig 9:**
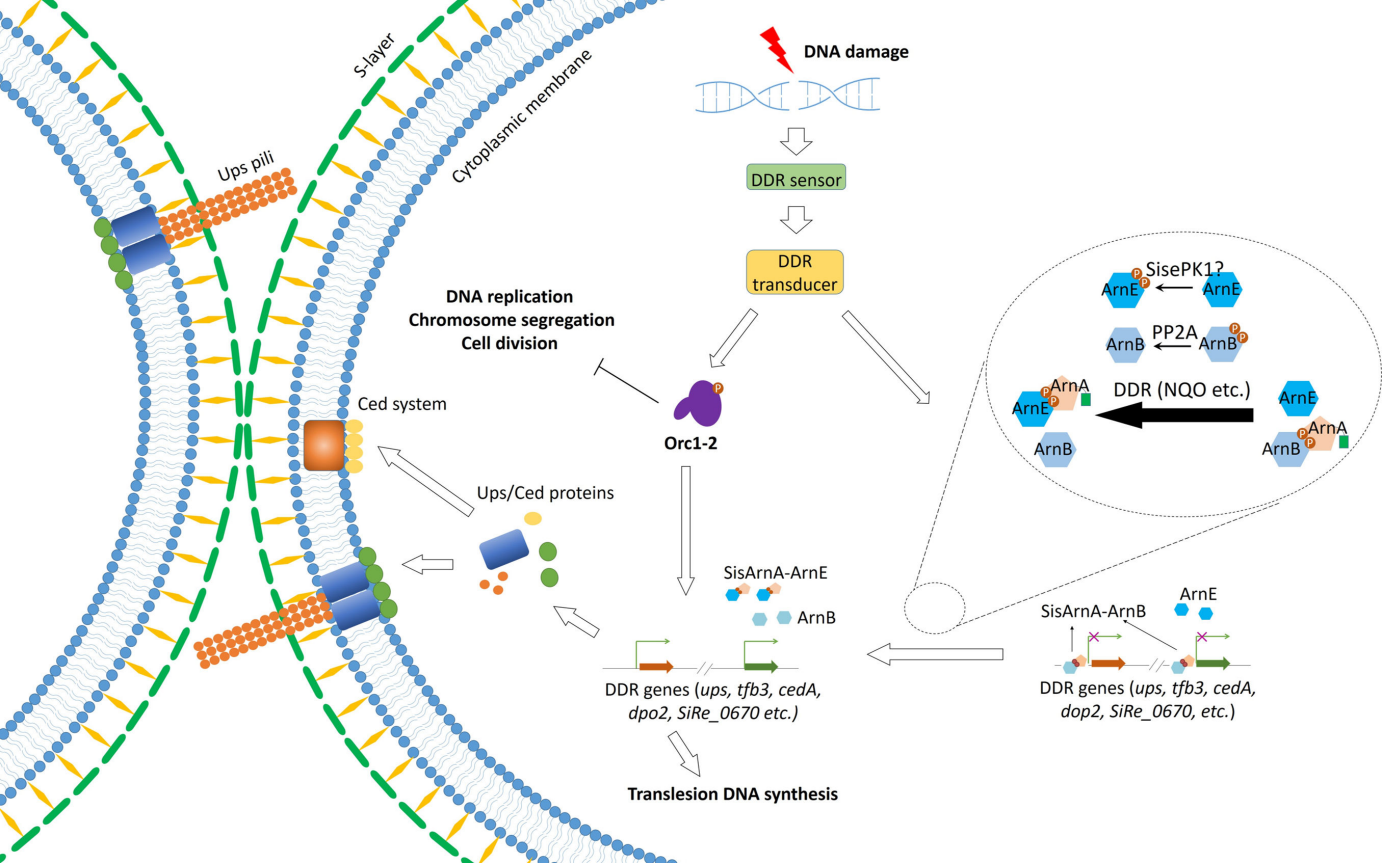
A proposed model of the DNA damage response (DDR) network in *S. islandicus*. DNA lesions, such as double-stranded breaks, ignite the DNA signal transduction network which needs certain DDR sensors and transducers. The previously established pathway is dependent on Orc1-2 which mediates the up-regulation of the genes participating in DNA exchange/repair and down-regulation of the genes involved in DNA replication, chromosome segregation, and cell division (15). Another pathway revealed from this study is regulated via phosphorylation. SisArnA-ArnB interaction mediated by SisArnB phosphorylation represses a number of DDR genes. In the presence of DDR, the phosphorylation of SisArnB, a repressor, is eliminated by the phosphatase PP2A, resulting in decreased SisArnA-ArnB interaction. At the same time, the SisArnA-ArnE interaction, which might be mediated by SisePK1 phosphorylation, is enhanced resulting in further derepression of SisArnA-ArnB. By Orc1-2 and phosphorylation-mediated processes, the DDR genes encoding for proteins in DNA exchange/repair are up-regulated, ensuring genome integrity maintenance and cell survival. In addition, Orc1-2 may also be regulated by protein phosphorylation.

In conclusion, we establish that *S. islandicus* FHA domain protein SisArnA is a global repressor of various genes, many of which were involved in DDR. Based on our results, we propose a model for DDR in *S. islandicus* (Fig. 9). In the presence of DDR, in addition to the Orc1-2-centered regulatory network, certain signals may induce a Arn-dependent regulation mediated by protein phosphorylation. The inhibition of a number of DDR genes is mediated by SisArnA-ArnB interaction under normal growth conditions. When DNA damage occurs, the interaction of SisArnA-ArnB is reduced by PP2A-mediated dephosphorylation of SisArnB, whereas SisArnA-ArnE interaction is enhanced via phosphorylation of SisArnE possibly by SisePK1. Then, the repression of the DDR genes by the SisArnA-ArnB complex is removed by ArnB dephosphorylation, or the transcription of the DDR genes is stimulated through SisArnA-ArnE interaction, facilitating DNA exchange/DNA repair *in vivo* and ensuring cell survival.

## MATERIALS AND METHODS

### Strains and growth conditions

*Saccharolobus islandicus* strain REY15A (E233S) (Δ*pyrEF*Δ*lacS,* Table S1) (hereafter, E233S) and its transformants were cultured as described previously (18).

### Plasmid construction

#### Construction of plasmids for gene knockout by the CRISPR-Cas system

The plasmids for the gene knockout were constructed based on the vector pGE (from Prof. Qunxin She’s lab) (49). Two complementary ssDNA of the protospacers (40 bp) within the target genes were synthesized by BGI (Beijing Genomics Institute, Beijing, China) and annealed to each other. The resulting protospacer DNA was inserted into pGE between two repeat sequences, yielding pGE-Sp. The L-arm and R-arm for recombination to delete the target gene were amplified and joined via splicing by overlap extension PCR and inserted into the *Sph*I and *Xho*I sites of pGE-Sp after the restriction enzyme digestion and purification. The sequences of PCR primers are listed in Table S2.

#### Construction of plasmids for protein expression

The plasmids for expression of *S. islandicus* ArnA, ArnB, and ArnE using pSeSD (50) were as described previously (18). The vectors pET15b and pET22b were applied for the construction of the plasmids expressing SisArnA and C-terminal His-tagged ArnB and ArnE, respectively, in *E. coli*. The genes were amplified using their corresponding primers (Table S2). The PCR products were digested with their corresponding restriction enzymes and ligated into the same restriction sites in pET15b or pET22b.

### DNA damage agents sensitivity assay

For drug treatment in a liquid medium, the strains were inoculated and cultured to the logarithmic phase for three times before treatment. DNA damage agent 4-nitroquinoline 1-oxide (NQO, 3 µM) was added into a 30-mL aliquot of each strain at an OD_600_ = 0.2. These flasks were then incubated at 75°C. The OD_600_ values were measured every 6 or 12 h thereafter. The growth curves were derived based on data from three biological repeats. For the cell viability assay, cultures treated with NQO for 6 h were collected and diluted properly. Each aliquot of 500–1,000 cells was spread on plates and cultivated at 75°C for 5–7 days. The survival ratio was calculated by the number of colonies from NQO-treated sample divided by that from the untreated sample. The data were obtained from three independent experiments.

### Flow cytometry

The *Saccharolobus* cells were collected and treated with 70% ethanol. Flow cytometry analysis was performed as described previously (51).

### Microscopy analysis and cell aggregation formation assay

The wild-type strain and Δ*SisarnA* were cultivated at an initial OD_600_ = 0.04. After overnight cultivation, the samples at the early log phase (OD_600_ = 0.2) were taken and observed under a Nikon Eclipse80i Microscope (Nikon Corporation, Tokyo, Japan) or Nikon Eclipse Ti-E Inverted Microscope. The cell diameters were measured by the software NIS-Elements AR 3.1. Cell aggregation ratio was calculated from images taken with the inverted microscope. Cell clusters containing more than three cells were considered cell aggregation. More than 500 cells were counted for each sample. One-tailed Student’s *t*-test was used for statistical analysis on the software GraphPad Prism 5.

### Promoter activity assay

The promoter activity of DDR genes was assayed according to the previous study (52). Briefly, the gene of *S. solfataricus* β-glycosidase (SsolacS) was amplified from its genome and inserted into the multiple cloning sites (MCS) of pSeSD. The promoter regions (200 bp upstream of start codons) of target DDR genes were amplified and inserted into the *Sph*I /*Nde*I site of pSeSD-SsolacS to replace the arabinose promoter, yielding the reporter plasmids. The plasmids were transformed into *S. islandicus* E233S or Δ*SisarnA* individually, and three single colonies were picked for each strain. They were grown in MTSV (Mineral salt medium + Typtone + Sucrose + Vitamin) for 6 h in the presence or absence of NQO. Cells were collected and lysed by sonication. The β-glycosidase activity in the cell extracts of different *S. islandicus* strains was determined using substrate β-nitrophenyl-*b*-d-galactopyranoside (ONPG) as described previously (52). One-tailed Student’s *t*-test was used for statistical analysis on the software GraphPad Prism 5.

### RT-qPCR

Total RNA was isolated by the Trizol agent. Briefly, cells were resuspended with 1 mL Trizol and vortex. After incubation for 5 min at room temperature, 200 µL chloroform was added to remove proteins. Then, RNA was isolated from the mixture by centrifugation with 13,000 × *g* for 15 min at 4°C and precipitated with an equal volume of isopropanol. RNA was washed with 70% ethanol and dissolved into RNase-free water. Reverse transcription was performed using EvoM-MLV RT Mix Kit (Accurate Biotechnology Cp., Ltd, Hunan, China). Firstly, genomic DNA was removed by incubating the RNA with five gDNA Clean Reaction Mix at 42°C for 2 min. First-strand cDNAs were then synthesized at 37°C for 15 min in a mixture containing EvoM-MLV RTase, RNase inhibitor, dNTPs, oligo dT(18T) Primer, and Random 6 mers Primer, followed by 85°C for 5 s. The mRNA levels of DDR genes were estimated by the cDNA sample using SYBR Green Premix Pro Taq HS qPCR Kit (Accurate Biotechnology Cp., Ltd, Hunan, China). The primers for qPCR were listed in Table S2. PCR was performed in CFX Connect Real-Time PCR Detection System (Bio-Rad, Hercules, CA, USA) with the following steps: denatured at 95°C for 30 s, 40 cycles of 95°C for 5 s, and 60°C for 30 s. The comparative Ct value of tbp cDNA was used as reference. One-tailed Student’s *t*-test was used for statistical analysis on the software GraphPad Prism 5.

### Protein purification

The plasmids for protein expression were transformed into *E. coli* BL21(DE3)-Codonplus-RIL. *Escherichia coli* harboring pET15b plasmids was cultivated in 1 L of LB medium containing ampicillin (100 µg/mL) and chloramphenicol (34 µg/mL). Proteins were induced by the addition of IPTG and incubation at 37°C for 4 h. The cells were collected by centrifugation at 10,000 × *g* for 3 min and disrupted by sonication in lysis buffer (50 mM Tris-HCl pH 8.0, 200 mM NaCl, 5% glycerol). The His-tagged proteins were purified with Ni-NTA agarose (Invitrogen) column and pooled for further purification by gel filtration with Superdex 200 increase 10/300 Gl column (GE Healthcare, Boston, MA, USA). Flag-tagged proteins were purified with Flag magnetic beads (GE Healthcare, USA) pre-equilibrated with buffer A (50 mM Tris-HCl pH 7.5, 150 mM NaCl, 5% glycerol). Proteins were eluted with Gly-HCl buffer (0.1 M Glycine pH 3.0 adjusted with HCl). The protein concentration was determined by the Bradford method with bovine serum albumin as the standard, and proteins were frozen by liquid nitrogen for storage at −80°C.

For protein purification in *S. islandicus*, the strains harboring corresponding expression plasmids were transferred in 1 L of *Sulfolobus* medium without sugar (MTV). d-arabinose was added into the culture when it reached OD_600_ = 0.2. The cells were cultivated for further 12–16 h before collected by centrifugation at 5,000 × *g* for 5 min. His-tagged and Flag-tagged proteins were purified as described above.

### Pull-down assay

For *in vitro* pull-down assay, indicated amounts of SisArnA (Flag-tag) and its putative interacting proteins (N-His) were mixed in buffer A and incubated at 65°C for 30 min. The mixture was then mixed with 100 µL of Ni-NTA beads (Life Technologies, Carlsbad, CA, USA) pre-equilibrated with buffer A and incubated at room temperature for 10 min by gently shaking. Unbound protein was removed by centrifugation at 3,000 × *g* for 5 min. After being washed with 400 µL of wash buffer (buffer A supplemented with 40 mM imidazole) for four times, the His-tagged protein and its putative interacted protein were eluted with 200 µL of elute buffer (buffer A supplemented with 250 mM imidazole). The fractions were analyzed by SDS-PAGE and Western blot. For the pull-down assay with phosphorylated proteins, SisArnB and SisArnE were incubated with the eukaryotes-like protein kinase SiRe_2056 (SisePK1) in a reaction containing 25 mM Tris-HCl pH 8.0, 25 mM NaCl, 5 mM MgCl_2_, 1 mM DTT, 10 mM ATP at 65°C for 30 min before applied for pull-down assay.

For the *in vivo* pull-down assay to detect the interactions of SisArnA with SisArnB and SisArnE, 300 mL of the complementation strains Δ*SisarnB*/pSeSD-Flag-ArnB and Δ*SisarnE*/pSeSD-Flag-ArnE was cultivated to OD_600_ = 0.2–0.3. The cells were treated with 3 µM NQO for 6 h before collection. The cells were resuspended in 20 mL buffer A for sonication. The soluble fractions (20 mL, input) were subjected to Flag magnetic beads for purification as described above. The eluted fractions (10 mL) were concentrated by 10 times. Twenty microliters of input and eluted samples was analyzed by Western blot with anti-Flag or anti-SisArnA antibodies. The gels were imaged with Amersham ImageQuant 800. The protein polyclonal antibody against SisArnA was raised in rabbits by Dai-An Biotechnology Co., Ltd (Wuhan, China) according to their standard procedure. The His-tagged SisArnA used for immunization was expressed and purified from *E. coli* BL21 as described above. The experiments were performed for three times independently. The ratios of SisArnA/SisArnB and SisArnA/SisArnE were quantified according to three independent experiments.

### Electrophoretic mobility shift assay

The fragments of the DDR gene promoters (200 bp upstream from the start codon of the DDR genes) were amplified using the corresponding primers and inserted into EcoRI and HindIII of pUC19, yielding pUC19 plasmids harboring DDR promoters. FAM-labeled substrates were obtained via PCR with the primers FAM-pUC19-MCS-F/pUC19-MCS-R and various pUC19 plasmids as templates. EMSA was performed in 20-µL reaction mixture containing 25 mM Tris-HCl pH 8.0, 25 mM NaCl, 5 mM MgCl_2_, 1 mM DTT, 5% glycerol, FAM-labeled dsDNA (200 bp), and various concentrations of the target proteins. The mixture was incubated at 37°C for 30 min and analyzed in 6% or 8% native PAGE as indicated. The gels were imaged by Amersham ImageQuant 800 (Cytiva, North Logan, UT, USA).

### *In vitro* kinase and phosphatase assay

The assays for protein phosphorylation and dephosphorylation *in vitro* were performed as described previously (17) with slight modification. Briefly, ATP was used for reaction, and phosphorylation signals were detected by Western blot with anti-pThr antibody (# 9381S; Cell Signaling Technology Inc., Danvers, MA, USA).

### Transcriptomic analysis of *SisarnA* deletion strain

The wild-type and *SisarnA* deletion strains were grown in an MTSV medium. When the cultures reached OD_600_ = 0.2–0.3, 3 µM NQO was added into the cultures. After incubation for further 6 h, the cells were collected by centrifugation (5,000 × *g*, 10 min). Total RNA extraction and RNA sequencing of the samples were carried out as described previously (53). The adjusted P-adjust (0.05) and log2 (fold change) of 1 were set as a threshold for significantly differential expression based on three independent cultures of each strain.

## Data Availability

The transcriptomic data reported in this study have been deposited in GEO and are accessible through GEO Series accession number GSE215078.

## SUPPLEMENTAL MATERIAL

Supplemental figures mbio.00942-23-s0001.docx Figures S1 to S8.

Tables S1 and S2 mbio.00942-23-s0002.docx Strains, vectors, and primers used in this study.

Table S3 mbio.00942-23-s0003.xls Transcriptomic data of E233S and ΔSisarnA in the presence and absence of NQO.

ASM does not own the copyrights to Supplemental Material that may be linked to, or accessed through, an article. The authors have granted ASM a non-exclusive, world-wide license to publish the Supplemental Material files. Please contact the corresponding author directly for reuse.
