# Supplementary material for: The FHA domain protein ArnA functions as a global DNA damage response repressor in the hyperthermophilic archaeon Saccharolobus islandicus
Source: mBio. 2023 Jun 30;14(4):e00942-23. doi: 10.1128/mbio.00942-23 (PMC10470591; doi:10.1128/mbio.00942-23)
Supplement: Supplemental figures — Figures S1 to S8. [file mbio.00942-23-s0001.docx]

Supplementary Figures for

**The FHA domain protein ArnA functions as a global DNA damage response repressor in the hyperthermophilic archaeon *Saccharolobus islandicus***

Zhichao Jiang, Zijia Lin, Qi Gan, Pengju Wu, Xuemei Zhang, Yuanxi Xiao, Qunxin She, Jinfeng Ni, Yulong Shen^#^, Qihong Huang^#^

CRISPR and Archaea Biology Research Center, State Key Laboratory of Microbial Technology, Microbial Technology Institute, Shandong University, 266237, Qingdao, China

^#^Correspondence: huangqihong@sdu.edu.cn, yulgshen@sdu.edu.cn


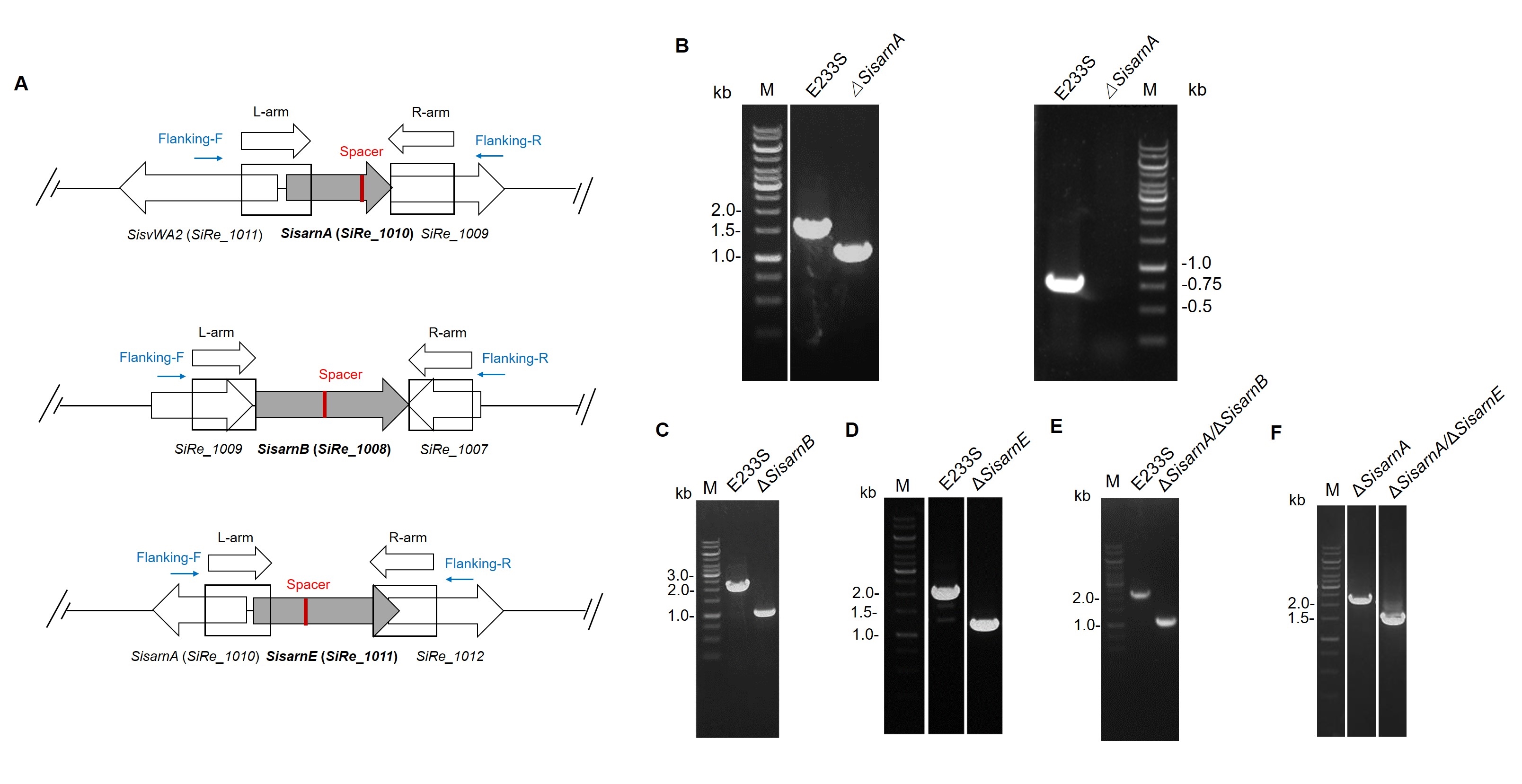


**Figure S1. Construction of the deletion strains of *S. islandicus,* Δ*SisarnA*, Δ*SisarnB*, Δ*SisarnE*, Δ*SisarnA/*Δ*SisarnB*, and Δ*SisarnA/*Δ*SisarnE.*** (**A**) Schematic for the knockout of *SisarnA, SisarnB* and *SisarnE.* The red indicates location of the protospacer for specific gene targeting. L-arm and R-arm, the up- and down-stream homologous recombination regions. The arrows in blue indicate flanking primers for PCR verification. (**B**) PCR verification of Δ*SisarnA* using SisArnA-Flanking-F/R primers (left) and gene specific primers (right). (**C**) to (**F**) Gene knockout verification by PCR using specific flanking primers for *SisarnB* and *SisarnE*, respectively.


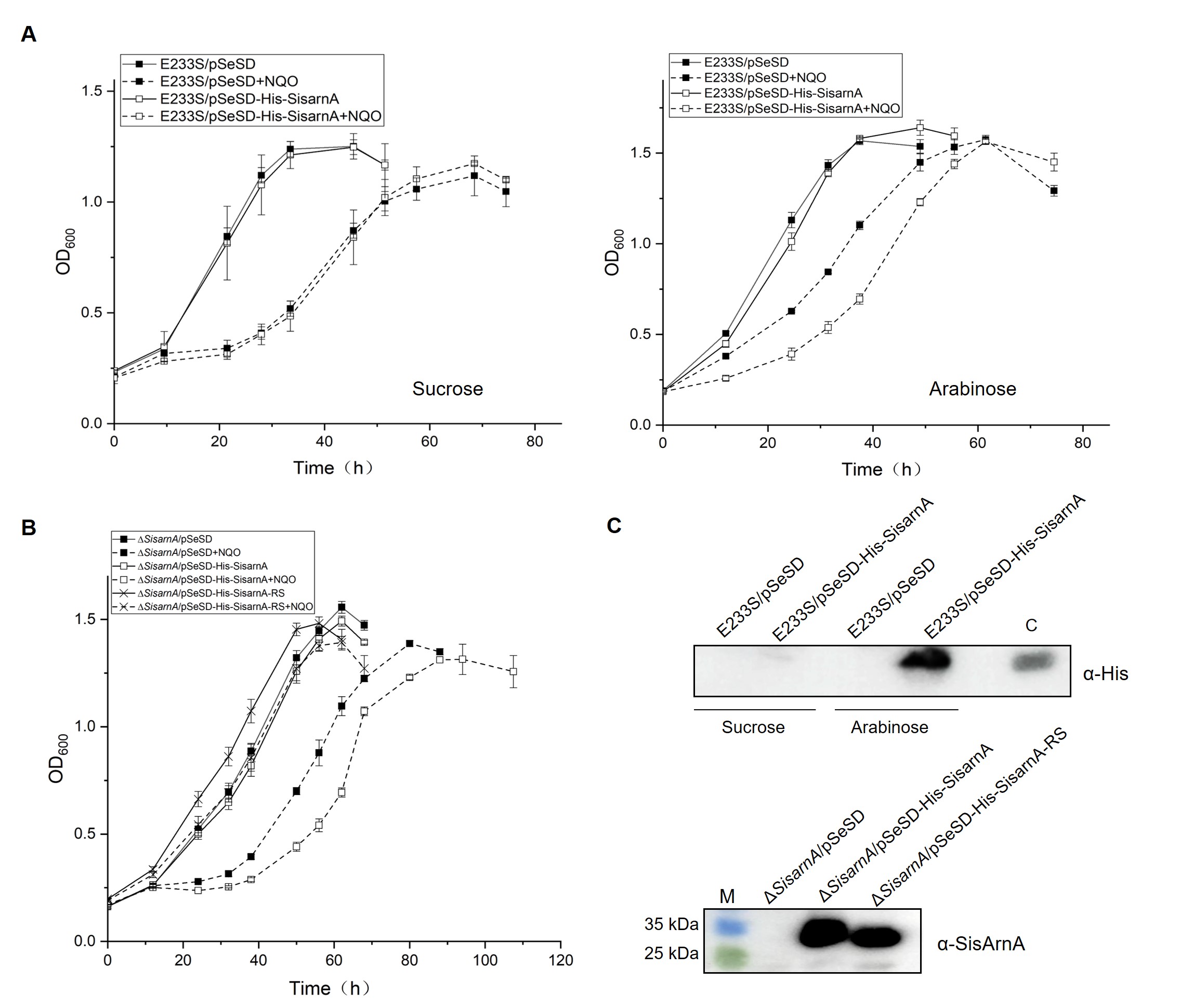


**Figure S2. Overexpression of the wild type SisArnA, but not its p-Thr binding deficient mutant, inhibited cell growth in the presence of NQO.** (A) Growth curves of E233S/pSeSD-His-SisArnA in sucrose-containing (left) and arabinose-containing medium (right) in the presence of 3 μM NQO. The wild type strain E233S harboring the empty vector pSeSD (E233S/pSeSD) was used as a control. (**B**) Complementation of wild type SisArnA, not its p-Thr binding deficient mutant, in Δ*SisarnA*, led to the sensitivity of *S. islandicus* to NQO. Growth curves of Δ*SisarnA*/pSeSD, Δ*SisarnA*/pSeSD-His-SisArnA and E233S/pSeSD-His-SisArnA-RS in arabinose-containing medium in the presence of 3 μM NQO. SisArnA-RS, p-Thr binding deficient mutant R134A/S148A. The values of OD_600_ were obtained from three independent cultures. Error bars indicate standard deviations. (**C**) Detection of His-SisArnA levels of the cells in (A) (upper panel) and (B) (lower panel). The samples in sucrose or arabinose medium at 12 h were taken for western blotting analysis. Antibody: α-His or α-SisArnA; C: His-SisArnA purified from *E. coli*; M: marker.


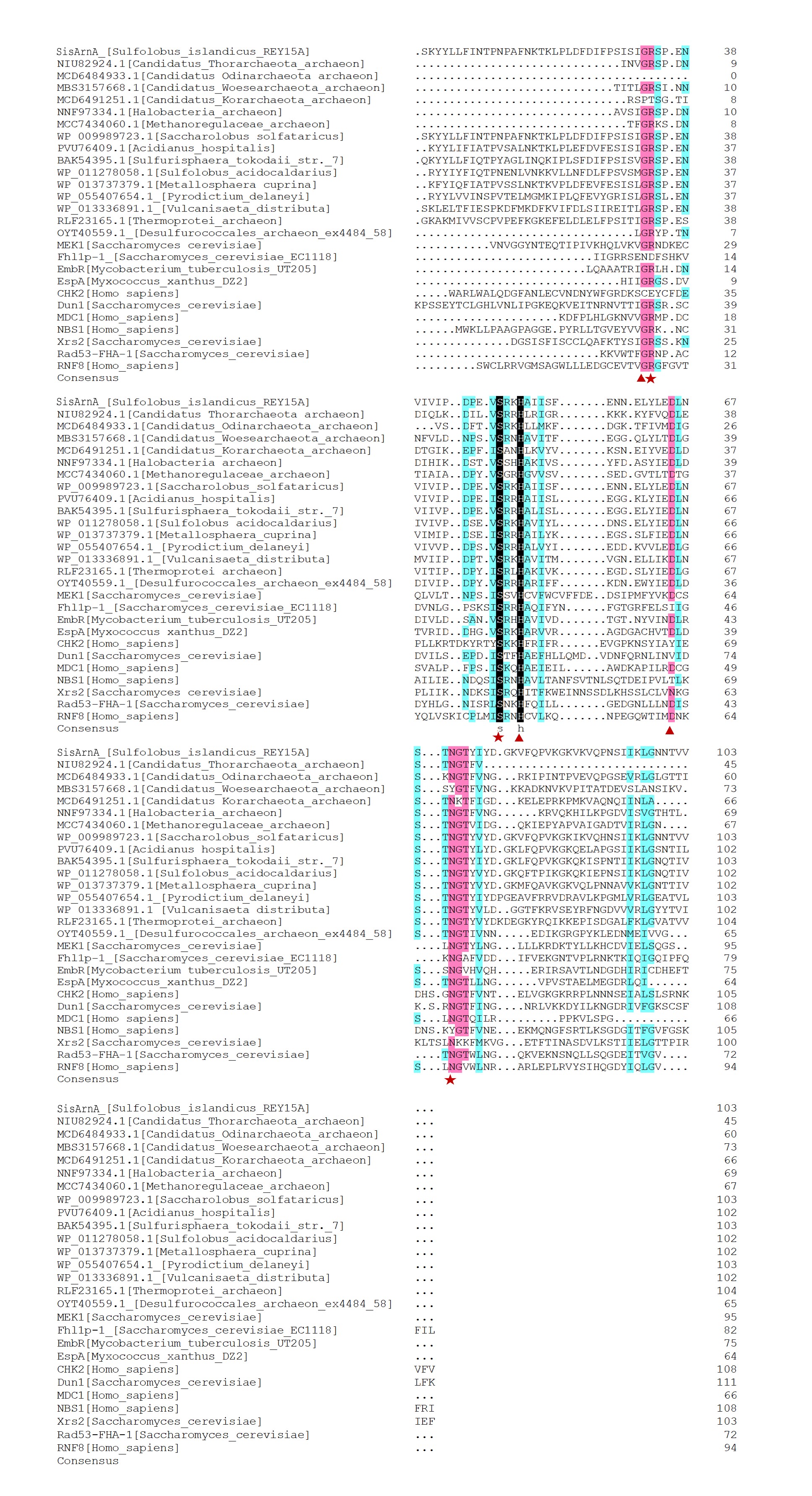


**Figure S3. Sequence alignment of the FHA domains from representative archaeal proteins and the eukaryotic proteins that are involving in DDR.** The sequences were obtained from NCBI. Archaeal FHA proteins were selected from representative archaeal phyla. Sequence alignment was performed using DNAMAN software. The residues colored in black, pink, and cyan, indicate 100% homology, 75%-100%, and 50%-75% homology, respectively. Red stars and triangles indicate conserved sites involved in binding to phosphorylated threonine (pThr) and the residue adjacent to the pThr, respectively.


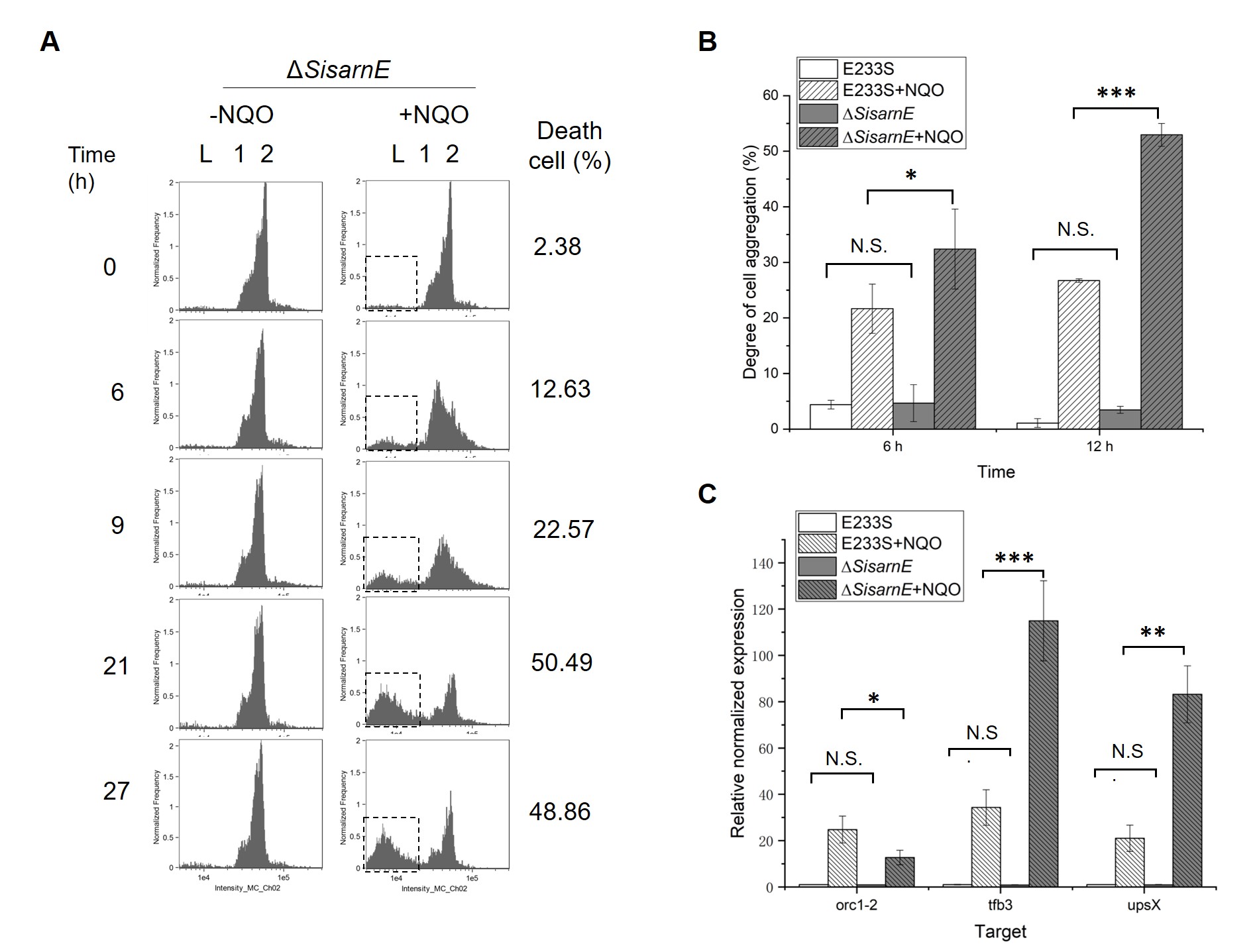


**Figure S4. Phenotypic analysis of Δ*SisarnE* compared with the wild type strain E233S in the presence (+) and absence (-) of the DNA damage agent NQO.** (**A**) Flow cytometry profiles. L, DNA-less cells; 1, cells containing one genome copy; 2, cells containing two genome copies. The ratios between the numbers of dead cells (boxed) and the total cell numbers at 6, 9, 21, and 27 h are indicated on the right in each NQO-treated sample. (**B**) Cell aggregation analysis. Quantification of the samples treated or untreated with NQO for 6h and 12 h. Aggregates containing more than three cells were counted. (**C**) RT-qPCR analysis of the transcripts of DDR genes (*orc1-2*, *tfb3*, and *upsX*) in E233S or Δ*SisarnE* cells treated or mock-treated with NQO for 6 h. The comparative Ct value of 16S rRNA was used as reference. The data was obtained from three independent experiments. T test was used for statistical analysis. Significant: * p<0.05, ** p<0.01, *** p<0.001, N.S. not significant.


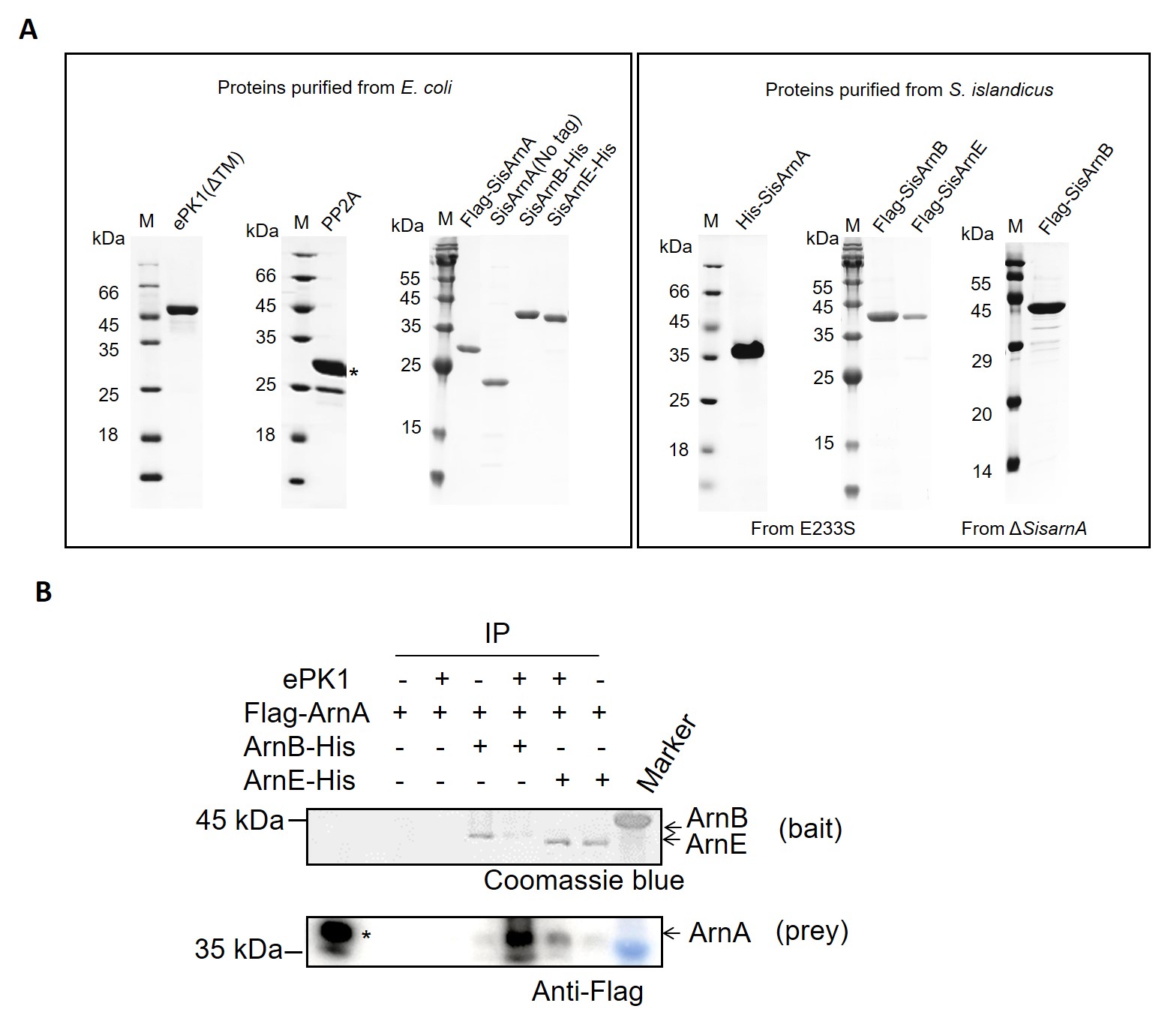


**Figure S5. *In vitro* analysis of the interactions between ArnA and ArnB or ArnE. (A)** SDS-PAGE profiles of the proteins purified from *E. coli* and *S. isandicus*. The asterisk indicates PP2A. **(B)** The interactions of SisArnB and SisArnE with SisArnA were stimulated by phosphorylation. His-tagged SisvWAs (35 μg) were firstly phosphorylated by incubation with ePK1 (SiRe_2056, 6 μg) at 60^o^C. Flag-SisArnA (30 μg) was then added into the reaction mixture. After incubation with Ni-NTA beads, the eluted samples were analyzed by western blot with anti-Flag antibody. The asterisk indicates the control signal of SisArnA purified from *E. coli*.


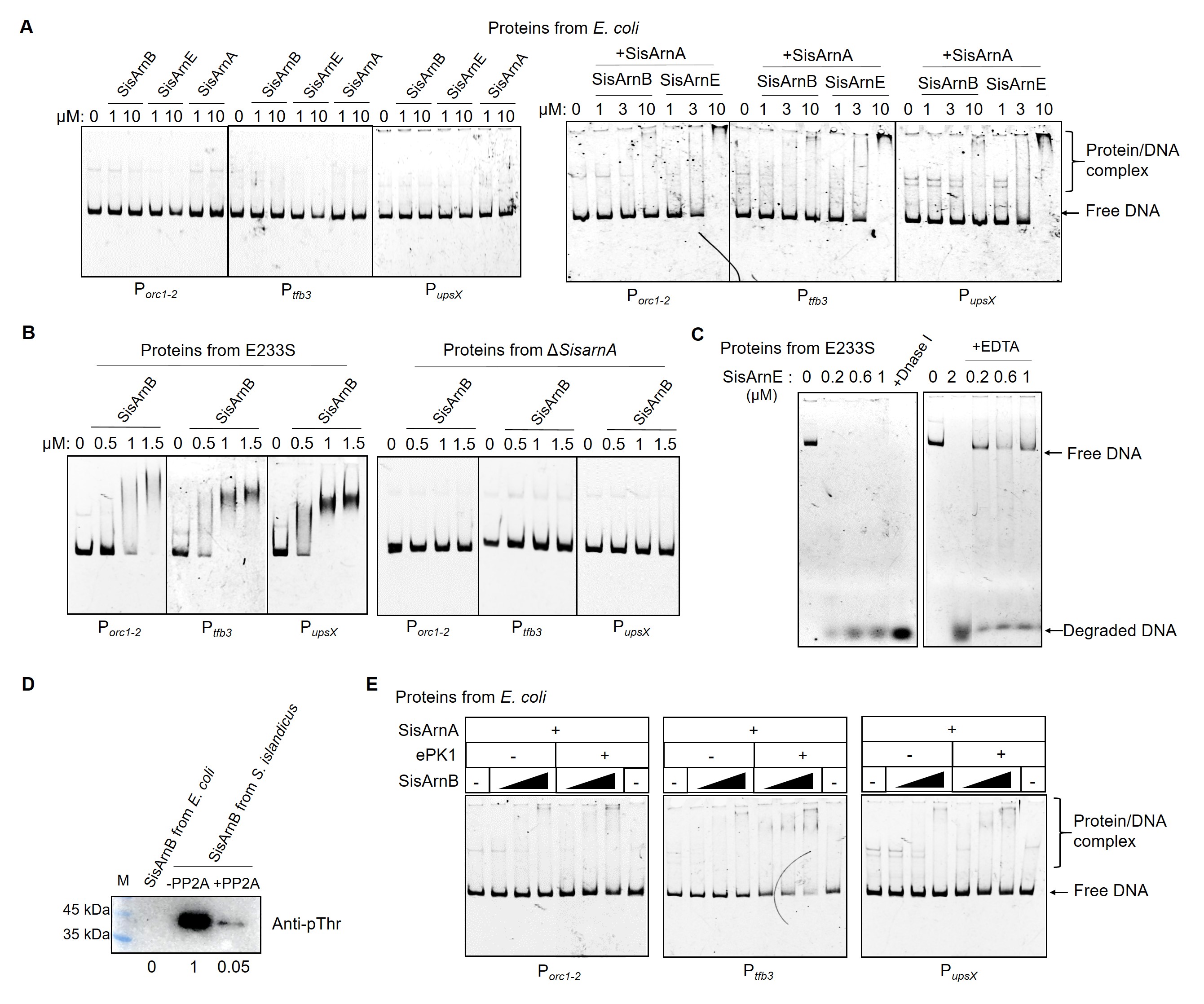


**Figure S6. Analysis of the DNA binding ability of SisArnA, SisArnB, and SisArnE.** (**A**) SisArnB or SisArnE in combination with SisArnA, but not individually, exhibited DNA binding activity. All proteins were purified from *E. coli.* EMSA was performed in a reaction mixture (20 μl) containing 500 bp FAM-labelled DNA (the promoter sequence of *orc1-2*, *tfb3, or upsX*) and the indicated amount of proteins at 37^o^C for 30 min. The samples were analyzed using 6% native PAGE. After electrophoresis, the gels were scanned using Amersham ImageQuant 800. (**B**) The SisArnB protein purified from Δ*SisarnA* could not bind the three promoters. EMSA was performed as that in (A) except that the SisArnB proteins purified from *S. islandicus* E233S or Δ*SisarnA* were used in the reaction with 200 bp FAM-labelled promoters as substrates. (**C**) The SisArnE sample purified from *S. islandicus* exhibited DNA degradation activity. The indicated concentrations of SisArnE was incubated with DNA (P*_tfb3_*) in the EMSA buffer with or without EDTA (1 mM) at 37^o^C. DNase I was used as a control. (**D**) SisArnB purified from *S. islandicus*, not *E. coli,* was phosphorylated. The SisArnB samples from *S. islandicus* was dephosphorylated by PP2A (SiRe_1009) at 60^o^C for 30 min in the reaction mixture as described in the Materials and Methods. The SisArnB samples (1 μM) purified from *E. coli* and *S. islandicus* and the dephosphorylated SisArnB were loaded onto SDS-PAGE and the phosphorylation signals were detected by western blot with anti-pThr antibody (CST No. 9381S). The relative intensity of signals are indicated at the bottom. M, marker. (**E**) Phosphorylated SisArnB exhibited slightly higher DNA binding activity than that of untreated SisArnB in the presence of SisArnA. After phosphorylation of SisArnB (1, 3, or 10 μM) by ePK1 (SiRe_2056, 0.5 μM), SisArnA and DNA was added into the mixture and incubated for further 30 min at 37^o^C. The samples were analyzed by 6% native PAGE.

**
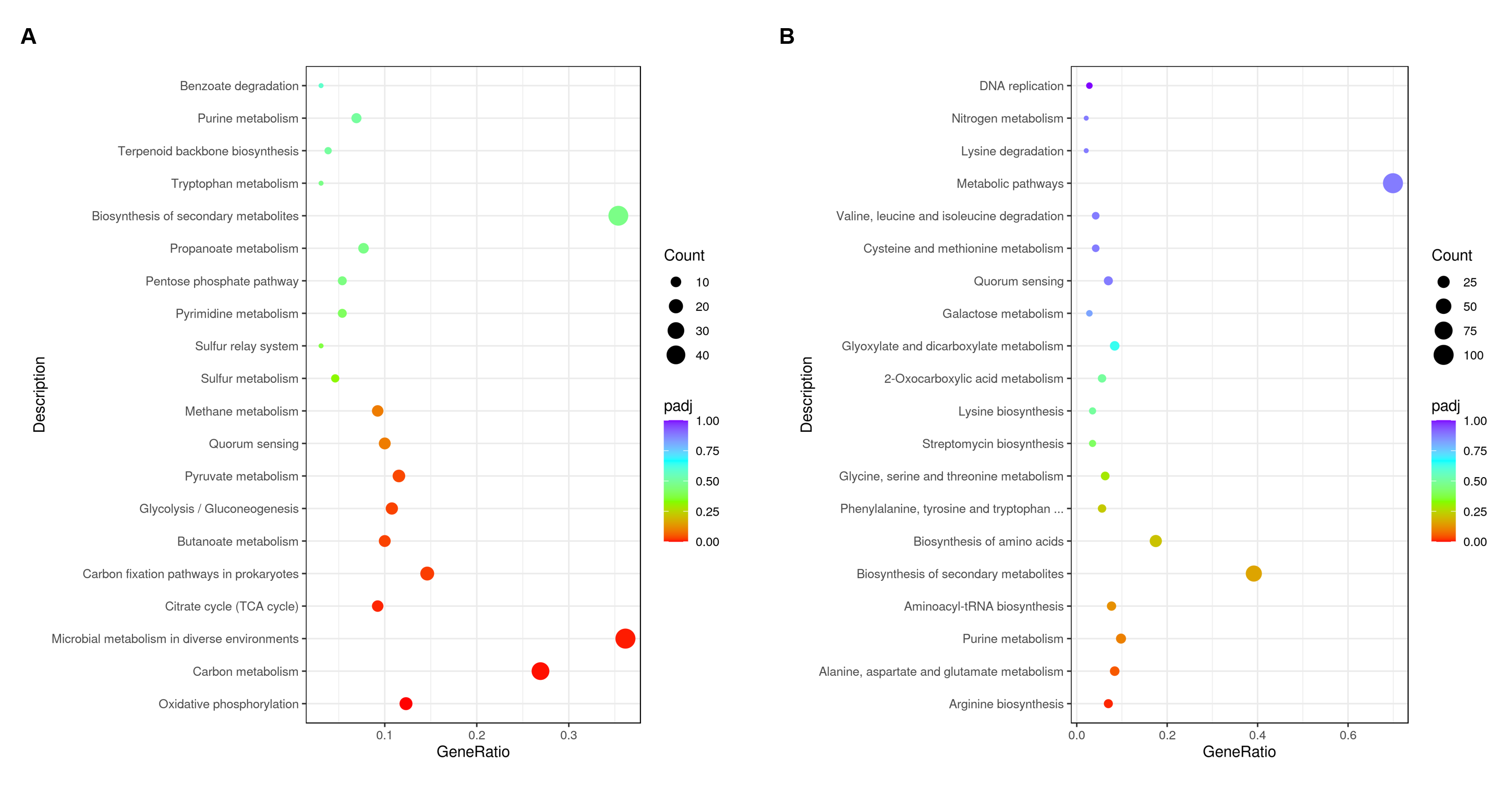
 Figure S7. KEGG enrichment of up- (A) and down-regulated (B) genes in Δ*SisarnA* compared with E233S.** The KOBAS software was used to test the statistical enrichment of differential expression genes in KEGG pathways. X-axis, the ratio of differentially expressed genes involved in a KEGG pathway to all differentially expressed genes. Y-axis, description of KEGG pathways. The size of circles indicate number of genes. Colors indicate padj values.


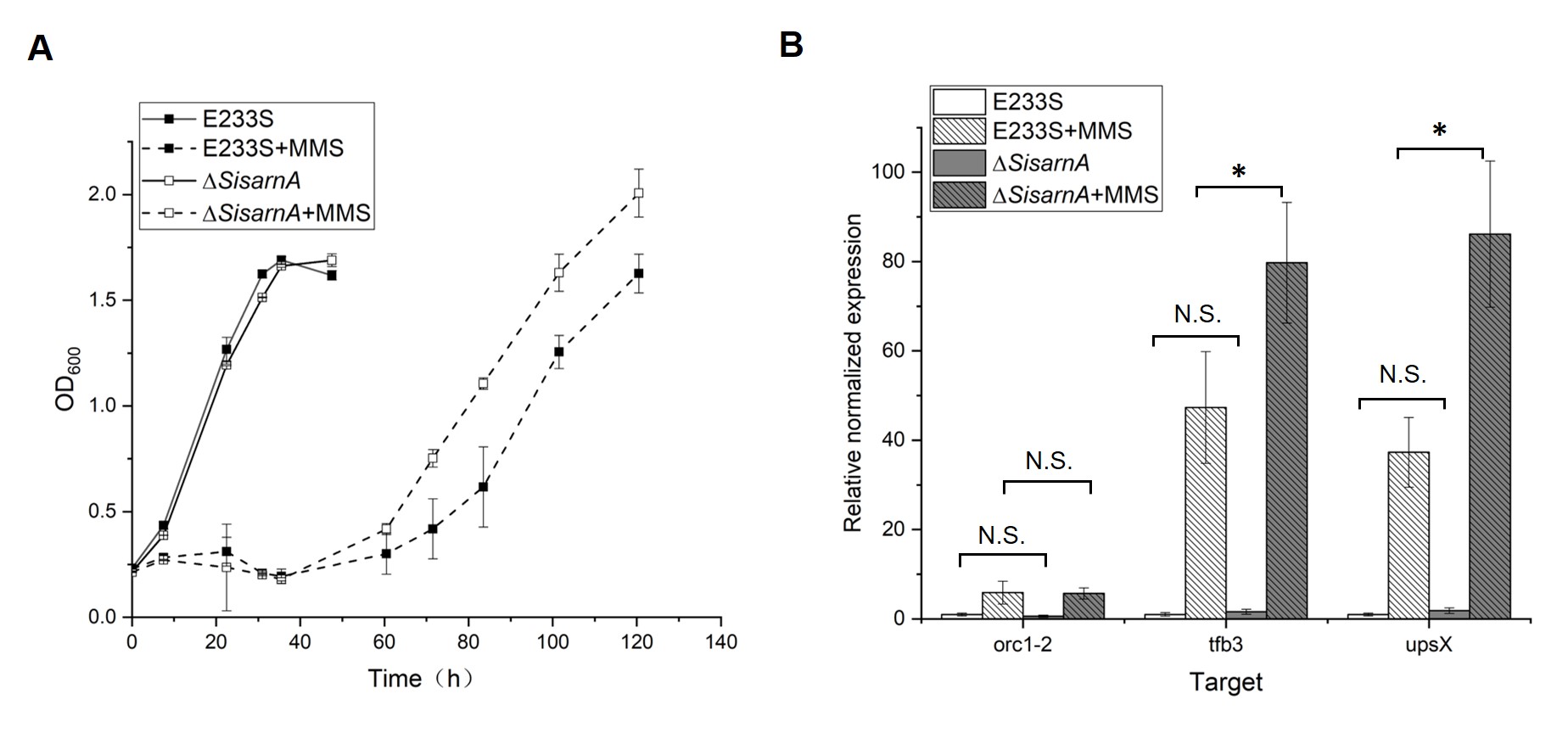


**Figure S8. Comparison of the cell growth and the transcription of the DDR genes between Δ*SisarnA* and E233S in the presence (+) and absence (-) of MMS.** (**A**) Growth curves. The cultures were grown to OD_600_~0.2 before the DNA damage agent MMS (2 mM) was added. The values of OD_600_ were obtained from three independent cultures. Error bars indicate standard deviations. (**B**) RT-qPCR analysis of the transcripts of the DDR genes. E233S or Δ*SisarnA* cells were treated with MMS for 6 h and their total RNA was extracted for RT-qPCR with corresponding qPCR primers of *orc1-2*, *tfb3*, and *upsX*. The data were obtained from three independent experiments. Error bars indicate standard deviations. T test was used for statistical analysis. Significant: * p<0.05, ** p<0.01, N.S. not significant.
