# Supplementary material for: The FHA domain protein ArnA functions as a global DNA damage response repressor in the hyperthermophilic archaeon Saccharolobus islandicus
Source: mBio. 2023 Jun 30;14(4):e00942-23. doi: 10.1128/mbio.00942-23 (PMC10470591; doi:10.1128/mbio.00942-23)
Supplement: Tables S1 and S2 — Strains, vectors, and primers used in this study. [file mbio.00942-23-s0002.docx]

Supplementary Tables for

**The FHA domain protein ArnA functions as a global DNA damage response repressor in the hyperthermophilic archaeon *Saccharolobus islandicus***

Zhichao Jiang, Zijia Lin, Qi Gan, Pengju Wu, Xuemei Zhang, Yuanxi Xiao, Qunxin She, Jinfeng Ni, Yulong Shen^#^, Qihong Huang^#^

CRISPR and Archaea Biology Research Center, State Key Laboratory of Microbial Technology, Microbial Technology Institute, Shandong University, 266237, Qingdao, China

^#^Correspondence: huangqihong@sdu.edu.cn, yulgshen@sdu.edu.cn

**Table S1. Strains and vectors used in this study**

| Strain or vectors | Properties | Source or reference |
| --- | --- | --- |
| *S. islandicus* E233S | *S. islandicus* REY15A Δ*pyrEF* Δ*lacS* | [Deng *et al.*, 2009](#_ENREF_44) |
| Δ*SisarnA* | *SisarnA* knockout in E233S | this study |
| Δ*SisarnB* | *SisarnB* knockout in E233S | this study |
| Δ*SisarnE* | *SisarnB* knockout in E233S | this study |
| Δ*SisarnA*/*SisarnB* | *SisarnA and* *SisarnB* double deletion in E233S | this study |
| Δ*SisarnA*/*SisarnE* | *SisarnA and* *SisarnB* double deletion in E233S | this study |
| E233S/pSeSD-His-SisArnA | His-SisArnA overexpression in E233S | this study |
| E233S/pSeSD-His-SisArnA-RS | His-SisArnA-R134A/S148A overexpression in E233S | this study |
| Δ*SisarnA*/pSeSD | *SisarnA* knockout strain harboring the empty vector pSeSD | this study |
| Δ*SisarnA*/pSeSD-His-SisArnA | His-SisArnA complementation in Δ*SisarnA* | this study |
| Δ*SisarnA*/pSeSD-His-SisArnA-RS | His-SisArnA-R134A/S148A complementation in Δ*SisarnA* | this study |
| E233S/pSe-Porc1-2/tfb3/upsA/upsE/upsX-lacS | E233S harboring a reporter plasmid pSeSD-lacS containing the promoters (200 bp) of *orc1-2, tfb3, upsA, upsE,* or *upsX* | this study |
| Δ*SisarnA*/pSe-Porc1-2/tfb3/upsA/upsE/upsX-lacS | Δ*SisarnA* harboring a reporter plasmid pSeSD-lacS containing the promoters (200 bp) of *orc1-2, tfb3, upsA, upsE,* or *upsX* | this study |
| Δ*SisarnB*/pSeSD-Flag-SisarnB | Flag-SisArnB complementation in Δ*SisarnB* | this study |
| Δ*SisarnE*/pSeSD-Flag-SisArnE | Flag-SisArnE complementation in Δ*SisarnE* | this study |
| pGE | *Sulfolobus*-*E. coli* shuttle vector containing mini-CRISPR and *pyrEF* for CRISPR-Cas based gene editing | [Li *et al.*, 2016](#_ENREF_31) |
| pGE-*arnA*KO | gRNA targeted *arnA* knock out vector | this study |
| pGE-*arnB*KO | gRNA targeted *arnB* knock out vector | this study |
| pGE-*arnE*KO | gRNA targeted *arnE* knock out vector | this study |
| pSeSD-His-SisArnA | N-terminal 6×His tagged *Sis*ArnA expression vector | this study |
| pSeSD-SisArnA-His | C-terminal 6×His tagged *Sis*ArnA expression vector | this study |
| pSeSD-SisArnA-RS-His | C-terminal 6×His tagged *Sis*ArnA-R134A/S148A expression vector | this study |
| pSeSD-Flag-SisArnB | N-terminal Flag tagged *Sis*ArnB expression vector | this study |
| pSeSD-Flag-SisArnE | N-terminal Flag tagged *Sis*ArnE expression vector | this study |
| pSe-Porc1-2/tfb3/upsA/upsE/upsX-lacS | Promoter of araS-SD of pSe-LacS was replaced by the promoter of *orc1-2/tfb3/upsA/upsE/upsX* (200 bp upstream of start codon) | this study |

**Table S2. Primers used in this study**

| Primers | Sequence (5’-3’)* |
| --- | --- |
| SisArnAKO-Spacer-F | AAGATGGATATTGAGGGAAATATATCGAAATCTAGGGGTAATT |
| SisArnA-Spacer-R | AGCAATTACCCCTAGATTTCGATATATTTCCCTCAATATCCAT |
| SisArnA-L-arm-*Sph*I-F | AAAC**GCATGC**CCTTTACCTACGTCGAAA |
| SisArnA-L-R-arm SOE-R | CATCTAAATTCATCATTCTTGTCCACTTGCTTGA |
| SisArnA-L-R-arm SOE-F | TCAAGCAAGTGGACAAGAATGATGAATTTAGATG |
| SisArnA-R-arm-*Xho*I-R | GTTT**CTCGAG**GTCTGGTCTGGGTAATTT |
| SisArnA-Flanking-F | GCTAGGTTAATGGTGAAGCTAACGG |
| SisArnA-Flanking-R | GGATCGTTCCACAATAATTGAAAGGC |
| SisArnBKO-Spacer-F | AAGGAAGGAATACACTTGCATTTTTTCGAAATAAGGTAACTTT |
| SisArnBKO-Spacer-R | AGCAAAGTTACCTTATTTCGAAAAAATGCAAGTGTATTCCTTC |
| SisArnBKO-L-arm-SphI-F | TCCG**GCATGC**GTATTATGGATTTAAGGT |
| SisArnBKO-L-R-arm SOE-R | CATCATTCCCTAAGCACATCACATCACCTAA |
| SisArnBKO-L-R-arm SOR-F | TTAGGTGATGTGATGTGCTTAGGGAATGATG |
| SisArnBKO-R-arm-XhoI-R | TGGC**CTCGAG**CATAATAACTAGGTATGAG |
| SisArnB-Flanking-F | GAGAGGGAATCACGAGAGTCCAATA |
| SisArnB-Flanking-R | GACATTTCATCTATAATTTCAACTGAAACTAG |
| SisArnEKO-Spacer-F | AAGGATGATCTATCTGCTAATTTCTTTAAGATTCTTTCATTAT |
| SisArnEKO-Spacer-R | AGCATAATGAAAGAATCTTAAAGAAATTAGCAGATAGATCATC |
| SisArnEKO-L-arm-SphI-F | TTGG**GCATGC**TACTTACAGTAGTTGTAGC |
| SisArnEKO-L-R-arm SOE-R | TAGTATCTAATCTCCAGTGGACTGTTATCAATC |
| SisArnEKO-L-R-arm SOR-F | GATTGATAACAGTCCACTGGAGATTAGATACTA |
| SisArnEKO-R-arm-XhoI-R | AAGC**CTCGAG**CTACCCTCCAATTTGAAA |
| SisArnE-Flanking-F | GCTGGATTAGGAGTATTGATGAAAAGG |
| SisArnE-Flanking-R | GCACAATGGCATTATCTTTAGGGATG |
| pGE-F | CCGAATTTATCTATCGCTTTTCTCTCTC |
| pGE-R | CGGACATATTTGCCCTAACAGATAAG |
| TBP-q-F | GTGGCAACAGTTACGTTAGAG |
| TBP-q-R | CCTTGGGCTGTTCTAATCTG |
| SiRe_1231-q-F | CAGAGAAGGAGGGATCACCA |
| SiRe_1231-q-R | TCCCATTGTAACCTCATCAGC |
| SiRe_1717-q-F | TGACGAGGGTATTTTGAGTGGT |
| SiRe_1717-q-R | TCCGATCACTTTCTTCGTTGAGT |
| SiRe_1878-q-F | ACTGGTTGGAGGGAGATCGA |
| SiRe_1878-q-R | ACAGTTCCGTCATTAGAAACCAGA |
| SiRe_1231-p-*Sph*I-F | CAAA**GCATGC**GAAATTGAAATGGGAGTAG |
| SiRe_1231-p-*Nde*I-R | GGGCTAA**CATATG**AACTTTTACCCCAATCTAAA |
| SiRe_1717-p-*Sph*I-F | TTAGCTGA**CATATG**ATGAAAACAATAACTTTTAT |
| SiRe_1717-p-*Nde*I-R | AGAT**GCATGC**ATTGTAACTCTTCCTCTCTG |
| SiRe_1881-p-*Sph*I-F | AGAT**GCATGC**ATTGTTCTATATTCGATAGC |
| SiRe_1881-p-*Nde*I-R | TTAGCTGA**CATATG**CTCTCACTATATCAAAATTT |
| SiRe_1879-p-*Sph*I-F | AGAT**GCATGC**TCATTTTCCCCATAGAGCCT |
| SiRe_1879-p-*Nde*I-R | TTCGGGG**CATATG**AATACTGAGTATTCAGAA |
| SiRe_1878-p-*Sph*I-F | ATAC**GCATGC**AAGACTATTATGATATAGC |
| SiRe_1878-p-*Nde*I-R | GGCA**CATATG**TTTTAATCCTTTATAAAAAG |
| pUC19-MCS-F | CCCAGTCACGACGTTGTAAAACGAC |
| pUC19-MCS-R | CACACAGGAAACAGCTATGACCATG |
| SisArnA-N-His-*Nde*I-F | TTAATTA**CATATG**CATCATCATCATCATCATACATGGAAATGTCCA |
| SisArnA-*Sal*I-R | GACT**GTCGAC**TCATTCTCCCACAATTCTC |
| pSeSD-F | TGGCGGTACATAGTGGTACATTAAAGTA |
| pSeSD-R | AAACCTTATGTTAAACTACGCCAGTAGG |
| SisArnA-N-Flag-*Nde*I-N-F | ATAGTAA**CATATG**GATTACAAGGATGACGACGATAAGACATGGAAATGTCCAGTT |
| SisArnA-*Xho*I-R | TATT**CTCGAG**TCATTCTCCCACAATTCTCAC |
| SisArnB-*Sac*I-F | AGTG**GAGCTC**GGTGACTCTTTCATTAAGAGTAG |
| SisArnB-*Sal*I-R | GGTG**GTCGAC**TCATTCCCTAAGCTTTCTAG |
| SisArnE-*BamH*I-F | CGT**GGATCC**GATGACTATTGCTATTAACCTTA |
| SisArnE-*Sal*I-R | ACTC**GTCGAC**TCATTGTCGCATCTTTCTAGTT |
| SisArnB-*Nde*I-F | TTTGCCG**CATATG**ACTCTTTCATTAAGAGTAGATA |
| SisArnA-N-His-*Nde*I-F | TTAATTA**CATATG**CATCATCATCATCATCATACATGGAAATGTCCA |
| SisArnA-*Sal*I-R | GTTT**GTCGAC**TCATTCTCCCACAATTCTC |
| SisArnB-N-Flag-*Xho*I-F | AATA**CTCGAG**ATGGATTACAAGGATGACGACGATAAGACTCTTTCATTAAG |
| SisArnB-*Nhe*I-R | CCCG**GCTAGC**TCATTCCCTAAGCTTTC |
| SisArnE-N-Flag-*Xho*I-F | TAAA**CTCGAG**ATGGATTACAAGGATGACGACGATAAGACTATTGCTATTAACC |
| SisArnE-*Nhe*I-R | GGCG**GCTAGC**TCATTGTCGCATCTTTCTA |
| SiRe_1231-*EcoR*I-F | GGC**GAATTC**AAATGTATAAATGAAATT |
| SiRe_1231-*Hind*III-R | GAT**AAGCTT**AACTTTTACCCCAATCTA |
| SiRe_1878-*EcoR*I-F | CCG**GAATTC**GGGAATATAATTTGTTAAA |
| SiRe_1878-*Hind*III-R | CCC**AAGCTT**ATATCCTCTTTTTAATCCTT |
| SiRe_1717-*EcoR*I-F | CGGG**GAATTC**AAATATCTAATTTTTTCTGCG |
| SiRe_1717-*Hind*III-R | CCGG**AAGCTT**ATGAAAACAATAACTTTTATGG |
| SiRe_1740-*EcoR*I-F | CCC**GAATTC**CTTACCAGACCTATATTCTTAA |
| SiRe_1740-*Sph*I-R | TCTT**GCATGC**CTTTTCTATTGTTTGTGCTA |
| SiRe_1719-*EcoR*I-F | TTCT**GAATTC**TTACTACATACAACTTCCCCG |
| SiRe_1719-*Hind*III-R | CGGG**AAGCTT**GTTAAGTCACTTTGACATTT |

* The restriction sites are in bold.
